# Characterization of disease-specific alterations in metabolites and effects of mesenchymal stromal cells on dystrophic muscles

**DOI:** 10.3389/fcell.2024.1363541

**Published:** 2024-06-14

**Authors:** Yuko Nitahara-Kasahara, Guillermo Posadas-Herrera, Kunio Hirai, Yuki Oda, Noriko Snagu-Miyamoto, Yuji Yamanashi, Takashi Okada

**Affiliations:** ^1^ Division of Molecular and Medical Genetics, Center for Gene and Cell Therapy, The Institute of Medical Science, The University of Tokyo, Tokyo, Japan; ^2^ Division of Cell and Gene Therapy, Nippon Medical School, Tokyo, Japan; ^3^ Division of Oral and Maxillofacial Surgical, Tokyo Women’s Medical School, Tokyo, Japan; ^4^ Division of Genetics, The Institute of Medical Science, The University of Tokyo, Tokyo, Japan

**Keywords:** Duchenne muscular dystrophy, mesenchymal stromal cells, metabolomics, cell therapy, *mdx* mouse

## Abstract

**Introduction:**

Duchenne muscular dystrophy (DMD) is a genetic disorder caused by mutations in the dystrophin-encoding gene that leads to muscle necrosis and degeneration with chronic inflammation during growth, resulting in progressive generalized weakness of the skeletal and cardiac muscles. We previously demonstrated the therapeutic effects of systemic administration of dental pulp mesenchymal stromal cells (DPSCs) in a DMD animal model. We showed preservation of long-term muscle function and slowing of disease progression. However, little is known regarding the effects of cell therapy on the metabolic abnormalities in DMD. Therefore, here, we aimed to investigate the mechanisms underlying the immunosuppressive effects of DPSCs and their influence on DMD metabolism.

**Methods:**

A comprehensive metabolomics-based approach was employed, and an ingenuity pathway analysis was performed to identify dystrophy-specific metabolomic impairments in the *mdx* mice to assess the therapeutic response to our established systemic DPSC-mediated cell therapy approach.

**Results and Discussion:**

We identified DMD-specific impairments in metabolites and their responses to systemic DPSC treatment. Our results demonstrate the feasibility of the metabolomics-based approach and provide insights into the therapeutic effects of DPSCs in DMD. Our findings could help to identify molecular marker targets for therapeutic intervention and predict long-term therapeutic efficacy.

## 1 Introduction

Duchenne muscular dystrophy (DMD) is an X-linked disorder triggered by primary abnormalities in the *DMD* gene that causes degenerative myopathy with secondary inflammation and necrotizing phase. Mutations in the dystrophin-encoding gene lead to dystrophin–glycoprotein complex deficiency in the sarcolemma, which leads to progressive degeneration/regeneration cycles in the striated muscle, manifesting as muscle weakness and eventual skeletal muscle atrophy (Ervasti et al., 1990; Campbell, 1995). In addition to the main symptoms of myopathy, patients often experience complications, such as endocrine metabolic disorders. As metabolic alterations also play a dominating influential role in the initiation and progression of various inherited or acquired diseases, abnormal metabolic function has been described as a part of the physiological challenges of DMD. Loss of dystrophin, the large membrane cytoskeletal protein, results in multiple systemic alterations, including extensive changes in energy production, in both patients (Boca et al., 2016; Srivastava et al., 2016; Spitali et al., 2018) and genetic animal models of DMD (Guiraud et al., 2015; Joseph et al., 2018; Lee-McMullen et al., 2019). Classically, creatine kinase (CK) released from leaked muscle tissue membranes, which is elevated in patients and animal models, is widely used as a clinical blood biomarker of DMD. However, its levels are highly variable and do not reflect the degree of muscle atrophy because they gradually decline and are no longer correlated with the severity of the disease.

Various studies have focused on metabolic alterations in the skeletal muscles (Dabaj et al., 2021; Merckx et al., 2022), cardiac muscles (Khairallah et al., 2007), brain (Tracey et al., 1996), and serum or plasma (Spitali et al., 2018; Xu et al., 2023) derived from patients with DMD, animal models, animals with Golden retriever muscular dystrophy (GRMD) (Abdullah et al., 2017), and *mdx* mice (Tsonaka et al., 2020).

MSCs are isolated from several organs, such as bone-marrow (Friedenstein et al., 1968), adipose tissue (Zannettino et al., 2008), amnion (Tsai et al., 2004), dental pulp (Zhang et al., 2008), peripheral blood (He et al., 2007), and cord blood (Oh et al., 2008) express several common cell surface antigenic markers, e.g., CD44, CD73, CD90, and CD105, and low levels of major histocompatibility complex class I molecules. They do not express hematopoietic markers CD34 or CD45 (Friedenstein et al., 1968). Dental pulp stem cells (DPSCs) obtained from decidual tooth tissue are a less invasive cell source, and demonstrated multipotency (Zhang et al., 2008) as well as high proliferative and immunosuppressive activities (Jo et al., 2007). DPSCs can also modulate immune effectors, thereby affecting crucial processes such as cell development, maturation, and function, as well as reactive T-cell responses (Ozdemir et al., 2016). Because regulating severe inflammation in dystrophic muscles could prolong the duration of therapeutic effects, DPSCs are also attractive candidates for cell-based strategies that target diseases with chronic inflammation, including DMD (Ichim et al., 2010). We previously demonstrated the therapeutic effects of systemic administration of DPSCs in model dogs and mice, i.e., preservation of long-term muscle function and slowing of disease progression (Nitahara-Kasahara et al., 2021). However, the mechanisms underlying the immunosuppressive effects of DPSCs, including abnormal skeletal muscle metabolism, have not been sufficiently characterized.

Currently, little is known regarding the effects of cell therapy on the metabolic abnormalities in DMD. In this study, we aimed to identify dystrophy-specific metabolomic impairments and assess therapeutic responsiveness to systemic DPSC administration via a comprehensive metabolomics-based approach using CE and liquid chromatography-mass spectrometry (LC–MS/MS). We also aimed to investigate whether metabolite monitoring is an appropriate component of the therapeutic evaluation of DMD.

## 2 Materials and methods

### 2.1 Animals

*Mdx* mice have a premature stop mutation in the exon 23 of the murine *Dmd* gene, which results in failure to translate dystrophin. These mice mimic various aspects of the human disease (Bulfield et al., 1984; Sicinski et al., 1989). C57BL/6-background *mdx* mice were developed by Dr. T. Sasaoka (National Institute for Basic Biology, Aichi, Japan) and were maintained in our animal facility. Age-matched and untreated male *mdx* littermates (P30 and P90, n = 3; P60, n = 6) and wild-type C57BL/6 mice (P30 and P90, n = 3; P60, n = 6) were used as controls in these metabolic studies. All mice remained healthy in appearance, activity, and body weight throughout the observation period. All experiments were conducted in accordance with the protocols described in the experimental protocols approved by the Ethics Committee for the Treatment of Laboratory Animals at the Nippon Medical School and Institute of Medical Science. The same male littermates were housed together in individually ventilated cages, with four to six mice per cage. Each group of mice was randomly assigned to a cage. All mice were maintained under a regular 12-h light/12-h dark diurnal lighting cycle with *ad libitum* access to food and water.

### 2.2 Culture and transplantation of DPSCs into mice and sampling

DPSCs were provided by JCR Pharmaceuticals (Hyogo, Japan). The cells were cultured in DMEM (Thermo Fisher Scientific) supplemented with 10% fetal bovine serum (Thermo Fisher Scientific) and 1% antibiotic-antimycotic solution (Wako Pure Chemical Industries) at 37°C in a 5% CO_2_ atmosphere. The animals *were* randomly divided into two groups: control and MSC-treated groups. Systemic delivery of DPSCs (8.0 × 10^5^ cells/100 μL of PBS) into *mdx* mice via the tail vein was conducted using four injections (treated-*mdx*, n = 3, each) administered at an interval of 1 week, beginning at 4–5 weeks of age (body weight [BW] > 10 g), as previously reported (Nitahara-Kasahara et al., 2021). Age-matched male WT and *mdx* mice (n = 3 each) were used as controls. At the ages of 30-, 60-, and 90 days (P30, P60, and P90), the animals were euthanized by cervical cord dislocation, and tissues were excised for histological and molecular analysis.

### 2.3 Histopathological analysis

Muscle samples were collected from the DPSC-treated mice and immediately frozen in liquid nitrogen-cooled isopentane. Subsequently, 8-μm-thick transverse cryosections were prepared from the skeletal muscles and stained with hematoxylin and eosin (H&E) using standard procedures. Tissue sections were visualized using the IX71 or IX83 microscope (Olympus, Tokyo, Japan). Quantification of nuclear infiltration and collagen-stained areas was performed using the HALO image analysis software (Indica Labs, Corrales, MN, USA). The nucleic area (%) was calculated by dividing the nuclear infiltration area by the total area.

### 2.4 Enzyme-linked immunosorbent assay (ELISA)

Serum CK levels were determined using an ELISA mouse kit (Cloud-Clone, Corp., TX, USA) according to the manufacturer’s recommendations.

### 2.5 Metabolomic analysis

Plasma and skeletal muscle samples were collected from young (P30) and adult (P60 and P90) *mdx* mice and age-matched WT mice (three male mice each group). The collected tissue samples (30 mg) were stored at −80°C until the assay was performed. Blood collected from the heart was centrifuged at 1,500 *g* for 15 min at 4°C. Plasma was isolated from the resulting supernatant.

Mouse plasma (50 µL) was added to a 450 µL methanol solution prepared to achieve a concentration of 10 µM of the pretreatment internal standard for capillary electrophoresis-time-of-flight mass spectrometry (CE-TOFMS). This mixture was added to 500 μL of chloroform and 200 μL of Milli-Q water and centrifuged at 2,300 *g* and 4°C for 5 min. After centrifugation, 400 μL of the aqueous layer was transferred to an ultrafiltration tube (Ultrafree MC PLHCC, HMT, centrifugal filter unit 5 kDa) and centrifuged (9,100 ×*g*, 4°C, 120 min) followed by ultrafiltration. The filtrate was dried and dissolved again in 50 μL of Milli-Q water for measurement. For liquid chromatography-time-of-flight mass spectrometry (LC-TOFMS) analysis, 300 μL of mouse plasma was added to 900 μL of formic acid-acetonitrile solution (1%) prepared as the internal standard (6 μM) and was centrifuged (2,300 ×*g*, 4°C, 5 min). Phospholipids were removed from the supernatant by solid-phase extraction and dried. For measurement, the dried sample was dissolved in 100 μL of 50% isopropanol solution (v/v).

The skeletal muscle sample (30 mg) added to 750 μL of 50% acetonitrile solution (v/v) was crushed using a crusher under cooling (1,500 rpm, 120 s × 3 times). The same volume of 50% acetonitrile solution (v/v) was added, and the sample was centrifuged at 2,300 *g* at 4°C for 5 min. The upper layer (400 μL) was transferred to an ultrafiltration tube (Ultra-free MC PLHCC, HMT, centrifugal filter unit 5 kDa) and was centrifuged (9,100 ×*g*, 4°C, 120 min), followed by ultrafiltration. The filtrated sample was dried and dissolved again in 50 μL of Milli-Q water for CE-TOFMS. For LC-TOFMS analysis, the skeletal muscle sample from the lower legs (30 mg) added in 500 μL of 1% formic acid-acetonitrile solution was crushed (1,500 rpm, 120 s × three times, and 1,500 rpm, 120 s × one time after addition of 167 μL Milli-Q water) using a crusher under cooling conditions and then centrifuged (2,300 × *g*, 4°C, 5 min). The supernatant was added to 500 μL of 1% formic acid-acetonitrile AMEOR-HMT-0096 and 167 μL of Milli-Q water to precipitate and stirred. Three tubes with a volume of 350 µL (NANOSEP 3 K Ω, PALL) were transferred to an ultrafiltration unit. The samples in the tubes were subjected to centrifugation (9,100 ×*g*, 4°C, 120 min) and ultrafiltration. After phospholipids in the mixture were removed using solid-phase extraction, the sample was dried and dissolved in 100 μL of 50% isopropanol solution (v/v) for analysis.

The mass spectrometers of the CE-TOFMS system (Agilent; Santa Clara, CA, USA) (Capillary: Fused silica capillary i.d. 50 μm × 80 cm, CE voltage: Positive, 27 kV; MS ionization: ESI Positive or ESI Negative; MS capillary voltage: 4,000 V, and MS scan range: m/z 50–1,000) and Agilent 1,200 series RRLC system SL (Column: ODS column, 2 × 50 mm, 2 μm; Column temp.: 40°C, Flow rate: 0.3 mL/min, Run time: 20 min, Post time: 7.5 min, Gradient condition: 0–0.5 min: B 1%, 0.5–13.5 min: B 1%–100%, 13.5–20 min: B 100%, MS ionization mode: ESI Positive, MS Nebulizer pressure: 40 psi, MS dry gas flow: 10 L/min, MS dry gas temp: 350°C, MS capillary voltage: 3500 V, MS scan range: m/z 100–1700) were operated in positive and negative electrospray ionization conditions. Three independent measurements were performed for each group.

Candidate compounds were assigned to 451 peaks in the plasma sample and 336 peaks in the skeletal muscle using the mass-to-charge ratio (*m/z*), migration time (MT), and retention time (RT) values of the substances in the HMT metabolite library (Human Metabolome Technologies Inc.) and the Kyoto Encyclopedia of Genes and Genomes (KEGG). For group comparisons, relative area ratios were calculated for each of the peaks corresponding to skeletal muscles and plasma, facilitating the identification of candidate compounds. Quantitative analysis was performed for the candidate compounds. To this end, the calibration curves incorporated peak areas corrected by internal standards, and concentrations were calculated via single-point calibration of 100 μM of substance (internal standard of 200 μM). Ingenuity pathway analysis (IPA; QIAGEN, CA, USA) was performed based on the quantified data from the plasma of each mouse group.

### 2.6 Statistical analyses

For each group, data were excluded when data acquisition was difficult due to weight loss, debilitation, or death or when there were concerns regarding peculiar values, mechanical errors in measurement, or external environmental influences because motor function and tissue structure could not be correctly assessed. Data are presented as the mean ± standard deviation (SD). Differences between the two groups were assessed using unpaired two-tailed t-tests. Multiple comparisons between three or more groups were performed using analysis of variance (ANOVA, n = 3, Tukey’s *post hoc* test). Statistical significance was defined as ^*^ *p* < 0.05, ^**^ *p* < 0.01, ^***^ *p* < 0.001, and ^****^ *p* < 0.0001 and was calculated using GraphPad Prism eight or 9 (GraphPad, La Jolla, CA, USA).

## 3 Results

### 3.1 *Mdx* mice repeatedly treated with DPSCs showed milder DMD disease phenotypes

**FIGURE 1:**
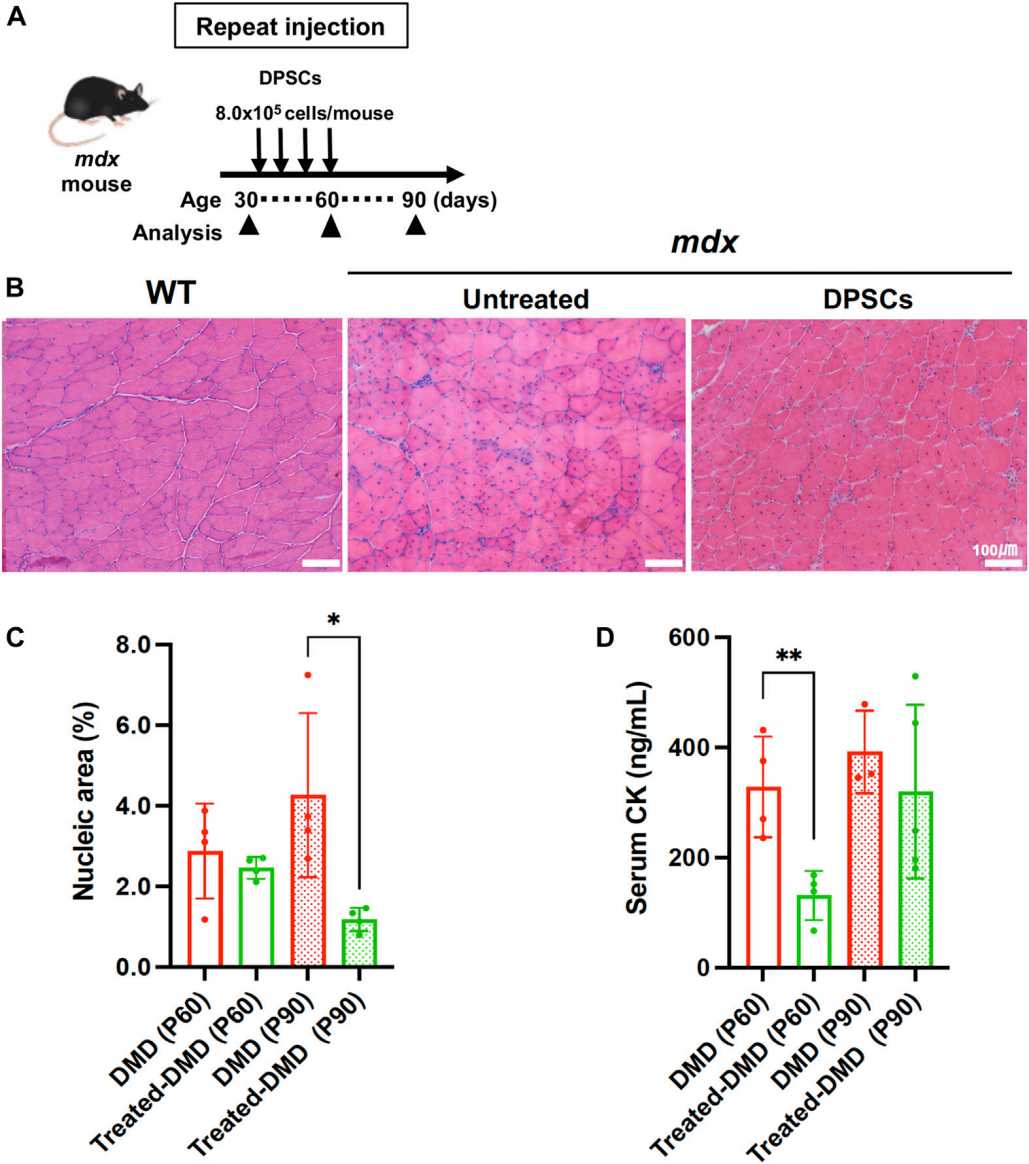
Systemic treatment of the *mdx* mice with DPSCs **(A)** Schematic representation of the repeated DPSC treatments of the *mdx* mice. **(B)** Hematoxylin and eosin (H&E) staining of the tibialis anterior (TA) muscle (original magnification, ×40, each) of untreated *mdx* mice and *mdx* mice treated four times with DPSC. Scale bars, 100 μm. **(C)** Quantification of the hematoxylin-positive area in the cross-section (% of total area) of the TA muscle of control *mdx* (DMD) mice and *mdx* mice treated four times DPSCs (Treated-DMD) at the age of 60 and 90 days (P60, P90, n = 4, each). Statistical differences between WT vs. DMD (^*^ *p* < 0.05, and ^**^ *p* < 0.01) are indicated; two-way ANOVA or multiple *t*-tests. **(D)** Serum creatine kinase (CK) levels in each group of mice; at the age of 60 and 90 days (P60, and P90) of control *mdx* (n = 4, and 3), and hDPSC-*mdx* (Treated-DMD; n = 4, each), using ELISA.

*Mdx* mice were repeatedly administered DPSCs via the tail vein (Figure 1A). The cross-section of the tibialis anterior (TA) muscle of the untreated *mdx* mice showed fibers with smaller (regenerating) and larger (hypertrophic) diameters, centrally nucleated fibers (CNFs), spread muscle interstitium, and cell infiltration interspersed in the muscle interstitium (Figure 1B). Histopathological findings observed in the repeatedly DPSC treated mdx mice included limited interstitial muscle area and nuclear infiltration. Quantitative analysis revealed a reduction in the nuclear inflammation area in the DPSC-treated TA muscle compared with that in untreated *mdx* mice (Figure 1C). In addition, high concentrations of circulating CK decreased temporarily after DPSC treatment in *mdx* mice (Figure 1D).

### 3.2 Metabolite variation on the dystrophic muscle with or without DPSC-treatment

We investigated the therapeutic effects of DPSC-treatment on the DMD pathology-specific metabolic abnormalities. To understand the biological responses to DPSC treatment or environmental changes in disease-specific features, we analyzed metabolic disturbances in plasma and TA muscles from WT (healthy), dystrophic *mdx*, and DPSC-treated *mdx* mice. Metabolic analysis by CE-TOFMS and LC-TOFMS revealed several substances in the plasma samples (451 metabolites; CE-TOFMS, cation: 195, anion: 95; LC-TOFMS, positive: 72, negative: 89, Supplementary Table S1) and skeletal muscle samples (460 metabolites; CE-TOFMS, cation: 212, anion: 126; LC-TOF-MS, positive: 85, negative: 37, Supplementary Table S2).

In the case of plasma samples, we used metabolic profiling to identify and quantitate a total of 165 metabolites, which revealed that changes in a total of 85 metabolites were statistically significant (*p* ≤ 0.05). The levels were either elevated or decreased in the comparison of untreated *mdx* mice versus WT (P30, 21 metabolites; P60, 26 metabolites; P90, 37 metabolites). Significant alterations were observed in 25 metabolites upon comparison of DPSC-treated *mdx* mice versus untreated *mdx* mice (P60, 11 metabolites; P90, 22 metabolites), and 31 metabolites were significantly changed upon the comparison of DPSC-treated *mdx* versus WT (P60, 27 metabolites; P90, 9 metabolites) (Supplementary Table S3).

Using global metabolic profiling, we identified and quantitated metabolites in the skeletal muscles. We found that changes in 62 metabolites were statistically significant (*p* ≤ 0.05), which either increased or reduced in the comparison of untreated *mdx* mice versus WT (P30, 23 metabolites; P60, 30 metabolites; P90, 37 metabolites). A total of 33 metabolites were significantly altered in the comparison of DPSC-treated *mdx* mice versus untreated *mdx* mice (P60, 11 metabolites; P90, 25 metabolites), and 50 metabolites were significantly changed in the comparison of DPSC-treated *mdx* mice versus WT (P60, 21 metabolites; P90, 41 metabolites) (Supplementary Table S4).

**FIGURE 2:**
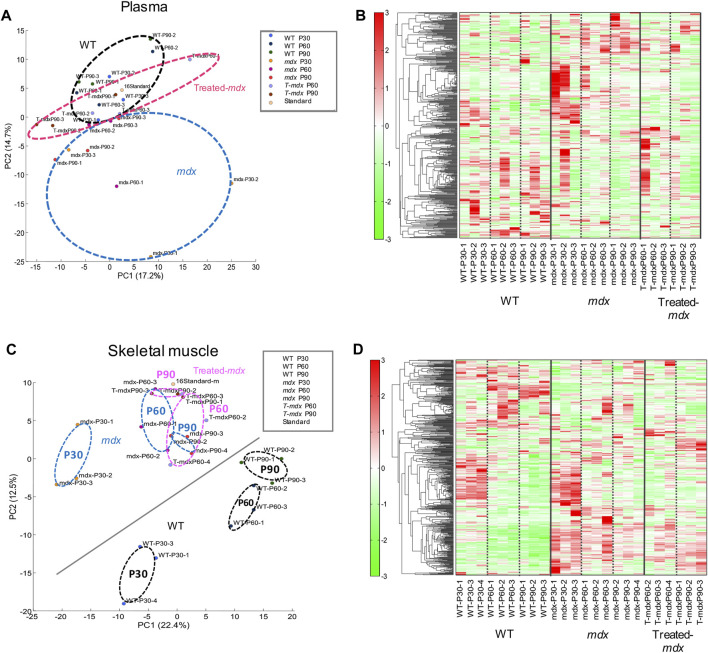
Principal component analysis (PCA) and differential concentration of metabolites A principal component analysis (PCA) plot **(A,C)** and heat map depicting hierarchical clustering analysis **(B,D)** of plasma **(A,B)** and the tibialis anterior (TA) muscles **(C,D)** derived from 30-, 60-, and 90-day-old (P30, P60, and P90) C57BL/6 mice (wild type, WT), untreated *mdx* mice (DMD), and DPSC-treated *mdx* mice (Treated-*mdx*). The PCA plot revealed significant separation among the mouse groups (n = 3). High and low concentrations of the metabolites are shown on a continuum from red to green.

Based on quantitative metabolite differences, principal component analysis (PCA) showed a clear separation between WT and dystrophic muscles (Figures 2A, B). The first and second PCA components explained 17.2% and 14.7% of the variation in plasma (Figure 2A) and 22.4% and 12.5% of the variation in the TA muscles (Figure 2B), respectively. In addition, DPSC-treated *mdx* mice showed an intermediate distribution between these groups in the PCA graph. While plasma samples showed negligible change with respect to the time course among mouse groups in the PCA results, we observed time-series variation in the analysis of data derived using the partial least-squares method (PLS) (Supplementary Figure S1). In the skeletal muscles, PCA analysis described clear time-series changes between 30 and 60–90 days for both WT and *mdx* mice. These data suggest that DPSC-treated *mdx* mice demonstrated a PCA distribution closer to that of WT mice than to that of untreated *mdx* mice in both plasma and skeletal muscle samples.

Considering the observed changes in metabolite levels, we next investigated how *mdx* and DPSC-treated mice exhibited metabolite imbalances compared to WT mice in the plasma and skeletal muscle. The heat map in Figures 2B, D shows the results of hierarchical clustering analysis (HCA). In HCA of skeletal muscle, WT and *mdx* mice showed notable differentiation consistent with PCA findings (Figure 2C), especially evident between the 30- and 60–90-day groups (Figure 2D). While less prominent than the differentiation between WT and *mdx* mice, we also detected some alterations in HCA patterns in DPSC-treated *mdx* mice compared to those in the untreated *mdx* mice, particularly at 60–90 days of age.

### 3.3 Metabolites that varied markedly in the dystrophic muscles with or without DPSC-treatment

The top and bottom 30 factor loadings annotated in the Human Metabolome Database (HMBD) containing PC1 and PC2 using PCA for the plasma samples (Table 1) as well as PC1 and PC2 for the skeletal muscle samples are listed in Table 2.

**TABLE 1:** Top and bottom 30 loading factors based on the principal component analysis (PCA) of plasma samples Based on the quantitative metabolic differences annotated in the Human Metabolome Database (HMBD), PCA was performed on plasma samples derived from each group, namely, wild-type, untreated *mdx* mice and DPSC-treated *mdx* mice. Factor loading top and bottom 30 metabolites sorted by PC1 (top, R > 0.727, *p* < 5.74E-05; bottom, R < −0.307, *p* < 1.45E-01) are listed in the upper two tables, whereas the corresponding top and bottom 30 metabolites sorted by PC2 (top, R > 0.678, *p* < 2.72E-04; bottom, R < −0.498, *p* < 1.33E-02) are listed in the bottom two tables.

Factor loadings (top 30)			PC1	
			R	*p*
Rank	ID	Compound name		
1	C_0128	*N*-Acetyllysine	0.920	1.94E-10
2	C_0024	GABA	0.884	9.98E-09
3	A_0075	6-Phosphogluconic acid	0.862	6.20E-08
4	A_0081	3′-CMP	0.851	1.36E-07
		2′-CMP		
5	C_0191	Glutathione (GSSG)_divalent	0.846	1.88E-07
6	A_0109	UDP-glucose	0.836	3.54E-07
		UDP-galactose		
7	C_0171	Malonylcarnitine	0.826	6.70E-07
8	A_0112	UDP-*N*-acetylgalactosamine	0.818	1.05E-06
		UDP-*N*-acetylglucosamine		
9	C_0022	3-Aminoisobutyric acid	0.813	1.41E-06
10	A_0049	3-Phosphoglyceric acid	0.811	1.52E-06
11	C_0198	*S*-Adenosylmethionine	0.802	2.48E-06
12	C_0093	Glu	0.794	3.71E-06
13	A_0048	2-Phosphoglyceric acid	0.778	7.70E-06
14	C_0195	NMN	0.776	8.48E-06
15	C_0049	Nicotinamide	0.772	1.00E-05
16	C_0046	Betaine aldehyde_+H_2_O	0.772	1.00E-05
17	C_0148	Kynurenine	0.763	1.42E-05
18	N_0046	*cis*-11-Eicosenoic acid	0.763	1.44E-05
19	C_0014	β-Ala	0.761	1.56E-05
20	P_0033	Ethyl arachidonate	0.759	1.73E-05
21	C_0190	Arg-Glu	0.755	1.99E-05
22	A_0083	UMP	0.752	2.27E-05
23	C_0085	4-Guanidinobutyric acid	0.747	2.74E-05
24	P_0016	Sphingosine	0.744	3.02E-05
25	C_0109	*S*-Methylmethionine	0.740	3.59E-05
26	C_0172	Pyridoxamine 5′-phosphate	0.739	3.75E-05
27	A_0090	GMP	0.734	4.49E-05
28	P_0068	α-Tocopherol acetate	0.731	4.98E-05
29	C_0197	*S*-Adenosylhomocysteine	0.729	5.33E-05
30	C_0088	Spermidine	0.727	5.74E-05

Factor loadings (bottom 30)			PC1	
			R	*p*
Rank	ID	Compound name		
30	P_0026	Deoxycorticosterone	−0.307	1.45E-01
		17α-Hydroxyprogesterone		
29	C_0005	Methylguanidine	−0.307	1.44E-01
28	C_0041	Val	−0.310	1.40E-01
27	P_0023	Linoleyl ethanolamide	−0.312	1.37E-01
26	N_0002	FA(13:0)	−0.315	1.33E-01
25	P_0072	1,2-Distearoyl-glycero-3-phosphocholine	−0.319	1.28E-01
23	N_0020	FA(16:3)-2	−0.326	1.20E-01
22	N_0015	FA(15:0)-2	−0.369	7.60E-02
21	P_0031	21-Deoxycortisol-1	−0.377	6.94E-02
20	N_0059	Leukotriene B4-1	−0.387	6.16E-02
19	C_0156	3-Hydroxykynurenine	−0.388	6.11E-02
18	P_0052	AC(16:2)-1	−0.389	6.04E-02
17	C_0050	Picolinic acid	−0.402	5.14E-02
16	A_0055	Phenaceturic acid	−0.423	3.96E-02
15	C_0118	Arg	−0.424	3.90E-02
14	P_0018	Estriol-1	−0.462	2.31E-02
13	N_0081	1-Palmitoyl-glycero-3-phosphoethanolamine	−0.470	2.06E-02
12	A_0013	2-Oxoisovaleric acid	−0.474	1.94E-02
11	A_0030	2-Oxoglutaric acid	−0.484	1.67E-02
10	C_0145	Trp	−0.500	1.29E-02
9	N_0067	FA(24:2)	−0.503	1.22E-02
8	A_0021	4-Methyl-2-oxovaleric acid	−0.540	6.51E-03
		3-Methyl-2-oxovaleric acid		
7	N_0039	FA(19:0)	−0.564	4.12E-03
6	P_0020	Progesterone	−0.575	3.31E-03
5	C_0165	Cystine	−0.577	3.13E-03
4	C_0121	Serotonin	−0.598	2.00E-03
3	N_0016	3-Hydroxytetradecanoic acid-1	−0.605	1.73E-03
		2-Hydroxytetradecanoic acid		
2	A_0060	*S*-Sulfocysteine	−0.643	7.09E-04
1	A_0003	Pyruvic acid	−0.742	3.28E-05

Factor loadings (top 30)			PC2	
			R	*p*
Rank	ID	Compound name		
1	P_0065	Hecogenin-2	0.809	1.72E-06
2	N_0066	FA(24:4)	0.808	1.80E-06
3	N_0057	FA(22:3)-1	0.791	4.19E-06
4	N_0056	FA(22:4)-2	0.773	9.57E-06
5	N_0051	*cis*-4,7,10,13,16,19-Docosahexaenoic acid	0.771	1.05E-05
6	N_0052	FA(22:5)-1	0.769	1.13E-05
7	N_0053	FA(22:5)-2	0.768	1.17E-05
8	N_0044	cis-11,14-Eicosadienoic acid-1	0.766	1.26E-05
9	N_0058	FA(22:3)-2	0.766	1.26E-05
10	N_0026	FA(17:2)	0.766	1.27E-05
11	P_0050	Stigmasterol-1	0.765	1.35E-05
12	N_0054	FA(22:5)-3	0.760	1.62E-05
13	N_0042	FA(20:3)	0.755	2.04E-05
14	N_0001	FA(12:0)	0.751	2.31E-05
15	N_0065	FA(24:5)	0.749	2.52E-05
16	N_0032	Linolenic acid	0.748	2.62E-05
17	N_0045	cis-11,14-Eicosadienoic acid-2	0.739	3.69E-05
18	N_0021	FA(16:2)-1	0.729	5.34E-05
19	N_0030	FA(17:0)-2	0.729	5.37E-05
		Heptadecanoic acid-2		
20	N_0031	Stearidonic acid	0.725	6.20E-05
21	P_0026	Deoxycorticosterone	0.717	8.06E-05
		17α-Hydroxyprogesterone		
22	N_0034	Oleic acid	0.716	8.37E-05
23	N_0027	FA(17:1)	0.706	1.14E-04
24	N_0022	FA(16:2)-2	0.705	1.18E-04
25	A_0024	2-Hydroxy-4-methylvaleric acid	0.702	1.31E-04
26	N_0033	Linoleic acid	0.700	1.41E-04
27	N_0055	FA(22:4)-1	0.696	1.61E-04
29	P_0048	5α-Cholestan-3-one-1	0.681	2.51E-04
30	N_0040	*cis*-5,8,11,14,17-Eicosapentaenoic acid	0.678	2.72E-04
		Abietic acid		

Factor loadings (bottom 30)			PC2	
Rank	ID	Compound name	R	*p*
30	C_0082	*N*-Ethylmaleimide_+H_2_O	−0.498	1.33E-02
29	A_0112	UDP-*N*-acetylgalactosamine	−0.498	1.33E-02
		UDP-*N*-acetylglucosamine		
28	A_0010	Glyceric acid	−0.499	1.31E-02
27	A_0025	Malic acid	−0.502	1.25E-02
26	A_0110	UDP-glucuronic acid	−0.506	1.16E-02
25	C_0061	6-Aminohexanoic acid	−0.522	8.83E-03
24	C_0027	Choline	−0.539	6.58E-03
23	C_0177	Thiamine	−0.541	6.35E-03
22	A_0090	GMP	−0.541	6.32E-03
21	C_0049	Nicotinamide	−0.542	6.26E-03
20	A_0104	UTP	−0.544	5.97E-03
19	A_0108	ADP-ribose	−0.545	5.87E-03
18	C_0160	Ergothioneine	−0.550	5.41E-03
17	A_0012	Fumaric acid	−0.550	5.36E-03
15	P_0071	Sphingomyelin(d18:1/16:0)	−0.563	4.15E-03
14	C_0081	Ectoine	−0.569	3.71E-03
13	C_0036	3-Amino-2-piperidone	−0.583	2.81E-03
12	A_0020	5-Oxoproline	−0.585	2.68E-03
11	A_0083	UMP	−0.587	2.55E-03
10	C_0122	Gluconolactone	−0.608	1.63E-03
9	C_0126	Phosphorylcholine	−0.608	1.62E-03
8	C_0143	Spermine	−0.610	1.54E-03
7	A_0016	Succinic acid	−0.632	9.21E-04
6	C_0090	*threo*-β-Methylaspartic acid	−0.635	8.55E-04
5	A_0095	UDP	−0.637	8.07E-04
4	A_0029	4-Acetamidobutanoic acid	−0.638	7.91E-04
3	C_0103	Imidazolelactic acid	−0.643	7.00E-04
2	C_0119	Guanidinosuccinic acid	−0.668	3.58E-04
1	C_0176	Glycerophosphocholine	−0.709	1.05E-04

**TABLE 2:** Top and bottom 30 loading factors based on the principal component analysis (PCA) of the skeletal muscles Based on the quantitative metabolic differences annotated in the Human Metabolome Database (HMBD), PCA was performed on the skeletal muscle derived from each group, namely, wild-type, untreated *mdx* mice, and DPSC-treated *mdx* mice. Factor loading top and bottom 30 metabolites sorted by PC1 (top, R > 0.512, *p* < 1.05E-02; bottom, R < −0.814, *p* < 1.30E-06) are listed in the upper two tables, whereas the corresponding top and bottom 30 metabolites sorted by PC2 (top, R > 0.636, *p* < 8.28E-04; bottom, R < −0.552, *p* < 5.16E-03) are listed in the bottom two tables.

Factor loadings (top 30)			PC1	
			R	*p*
Rank	ID	Compound name		
1	C_0181	Homocarnosine	0.903	1.56E-09
14	C_0220	Adenosine	0.740	3.60E-05
17	C_0167	Carnosine	0.719	7.66E-05
21	A_0135	NAD^+^	0.675	2.99E-04
25	P_0040	Campesterol	0.543	6.15E-03
27	A_0124	ATP	0.535	7.12E-03
28	C_0117	Serotonin	0.533	7.34E-03
30	A_0137	NADP^+^	0.512	1.05E-02

Factor loadings (bottom 30)			PC1	
			R	*p*
Rank	ID	Compound name		
30	C_0139	Ser-Ser	−0.814	1.30E-06
29	A_0052	Ribulose 5-phosphate	−0.816	1.18E-06
27	C_0076	Spermidine	−0.820	9.16E-07
26	C_0105	Taurocyamine	−0.824	7.66E-07
24	C_0032	Homoserine	−0.825	6.88E-07
23	A_0134	CMP-*N*-acetylneuraminate	−0.826	6.81E-07
22	C_0231	Glu-Lys	−0.826	6.51E-07
		Lys-Glu		
21	A_0043	Gluconic acid	−0.828	6.09E-07
19	P_0012	Sphinganine	−0.833	4.41E-07
18	A_0073	6-Phosphogluconic acid	−0.846	1.89E-07
17	C_0074	4-Guanidinobutyric acid	−0.850	1.47E-07
15	C_0135	Gly-Asp	−0.860	7.36E-08
13	A_0016	Ethanolamine phosphate	−0.862	6.00E-08
12	C_0097	*O*-Acetylhomoserine	−0.866	4.41E-08
		2-Aminoadipic acid		
10	C_0050	3-Guanidinopropionic acid	−0.872	2.93E-08
8	C_0130	*N*-Acetyllysine	−0.882	1.23E-08
		Ala-Val		
		Ile-Gly		
		Leu-Gly		
		Val-Ala		
7	A_0133	UDP-*N*-acetylgalactosamine	−0.892	4.91E-09
		UDP-*N*-acetylglucosamine		
6	A_0129	UDP-glucuronic acid	−0.896	3.39E-09

Factor loadings (top 30)			PC2	
			R	*p*
Rank	ID	Compound name		
1	C_0090	His	0.796	3.36E-06
2	C_0096	*N* ^6^-Methyllysine	0.788	4.87E-06
3	P_0072	Trilaurin-1	0.777	7.98E-06
4	C_0101	5-Hydroxylysine	0.763	1.47E-05
7	A_0039	Citric acid	0.738	3.87E-05
8	A_0037	3-Phosphoglyceric acid	0.734	4.42E-05
9	P_0073	Trilaurin-2	0.729	5.41E-05
11	C_0027	Pro	0.723	6.68E-05
12	C_0132	*N* ^6^,*N* ^6^,*N* ^6^-Trimethyllysine	0.720	7.31E-05
14	C_0008	Sarcosine	0.708	1.07E-04
15	C_0168	2′-Deoxycytidine	0.706	1.14E-04
16	C_0049	Hydroxyproline	0.706	1.15E-04
17	P_0074	Trilaurin-3	0.702	1.31E-04
18	C_0114	Citrulline	0.702	1.33E-04
19	C_0017	2-Aminoisobutyric acid	0.701	1.37E-04
		2-Aminobutyric acid		
20	A_0003	Pyruvic acid	0.697	1.54E-04
21	A_0018	2-Hydroxyglutaric acid	0.694	1.71E-04
22	C_0020	Ser	0.688	2.02E-04
23	C_0064	Trigonelline	0.688	2.04E-04
25	A_0047	Phosphocreatine	0.685	2.23E-04
28	C_0131	*N*_ω-_Methylarginine	0.639	7.73E-04
30	C_0144	ADMA	0.636	8.28E-04

Factor loadings (bottom 30)			PC2	
Rank	ID	Compound name	R	*p*
30	C_0289	Cysteine glutathione disulfide	−0.552	5.16E-03
29	C_0208	Ile-Gln	−0.554	4.95E-03
		Leu-Gln		
28	C_0216	Ile-Met	−0.559	4.54E-03
		Leu-Met		
		Met-Ile		
		Met-Leu		
26	C_0173	Ile-Val	−0.569	3.70E-03
22	A_0011	Isethionic acid	−0.585	2.68E-03
17	C_0087	Xanthine	−0.610	1.55E-03
13	C_0217	Phe-Val	−0.634	8.90E-04
12	C_0143	Ile-Ala	−0.644	6.83E-04
		Leu-Ala		
11	C_0073	Ile-Arg_divalent	−0.646	6.44E-04
10	C_0207	Glycerophosphocholine	−0.651	5.78E-04
8	A_0026	Uric acid	−0.671	3.33E-04
6	C_0111	Val-Gly	−0.691	1.87E-04
		*N*-Acetylornithine		
1	A_0010	2-Hydroxyvaleric acid	−0.808	1.81E-06

When we focused on the regions of the HCA map where a marked difference between WT and *mdx* mice was observed, we noticed that several amino acid metabolites were present in addition to HMBD. In terms of concentration, amino acid metabolites, such as Asn (asparagine), Asp, Lys, and Met, were accumulated in plasma samples (Supplementary Table S3), and Asn and Gln were abundant in the skeletal muscles of the *mdx* mice compared to those in the WT mice (Supplementary Table S4). Ser in plasma and Ile and Tyr in the skeletal muscle were lower than those in the WT. Accumulated amino acid metabolites in the *mdx* mice were downregulated in the DPSC-treated group, including Asn (ratio of *mdx* ver. WT P60, 1.5, *p* = 0.021; treated-*mdx* ver. *mdx*, 0.7; *p* = 0.046) and Met (ratio of *mdx* ver. WT P60, 1.27, *p* = 0.015; treated-*mdx* ver. *mdx*, 0.83, *p* = 0.035) in the plasma samples and Asn (ratio of *mdx* ver. WT P90, 1.49, *p* = 0.012; treated-*mdx* ver. *mdx*, 0.72; *p* = 0.023) in the skeletal muscles.

### 3.4 Identification of DMD and DPSC-treatment specifically altered metabolites

**FIGURE 3:**
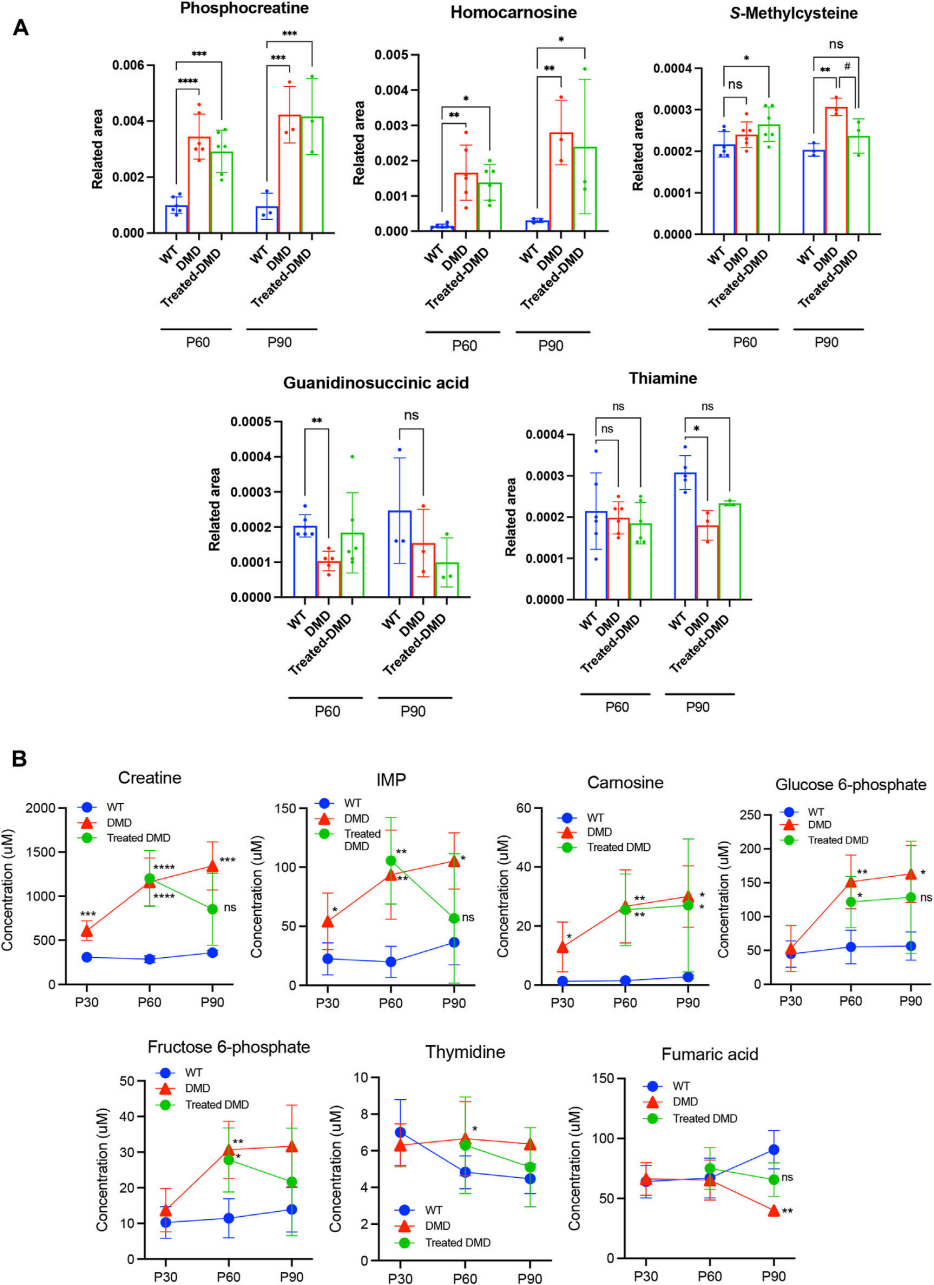
Altered metabolites in the plasma of dystrophic mice and effects of DPSC treatment **(A)** Quantitative analysis of metabolites altered in the plasma samples derived from 60- and 90-day-old (P60 and P90) C57BL/6 mice (wild type, WT), untreated *mdx* mice (DMD), and DPSC-treated *mdx* mice (Treated-*mdx*). Relative values of differentially expressed metabolites were described as fold changes compared to standard peaks. **(B)** Plasma levels of metabolite concentrations are described in 30–90-day-old mice (P30, P60, and P90). Statistical differences between WT vs. DMD (^*^ *p* < 0.05, ^**^ *p* < 0.01, ^***^ *p <* 0.001, and ^****^ *p <* 0.0001) and DMD vs. Treated-DMD (^*#*^ *p* < 0.05) are indicated; ns, not significant, two-way ANOVA or multiple *t*-tests. n = 3 for each group. Data are presented as the mean ± SD.

**FIGURE 4:**
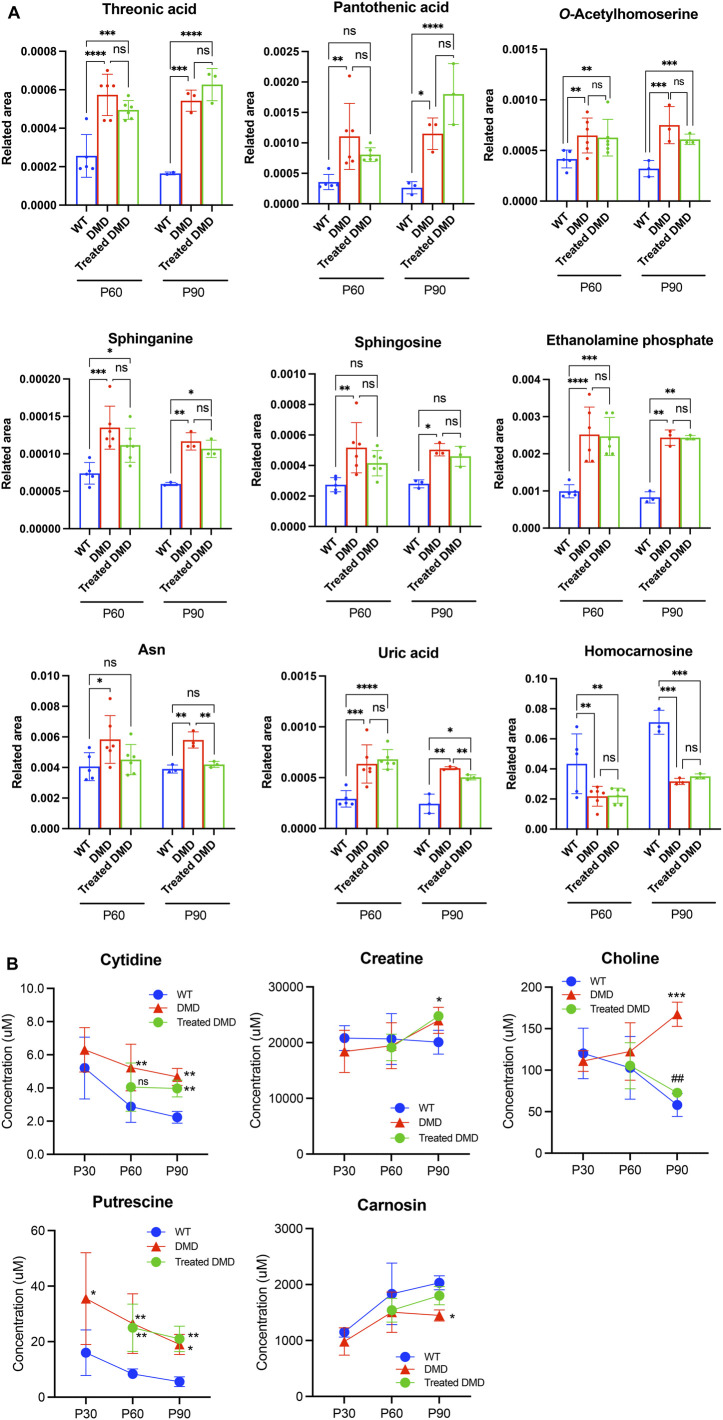
Altered metabolites in the skeletal muscle of dystrophic mice and effects of DPSC-treatment **(A)** Quantitative analysis of metabolites altered in the skeletal muscles derived from 60- and 90-day old (P60, and P90) C57BL/6 mice (wild type, WT), untreated *mdx* mice (DMD), and DPSC-treated *mdx* mice (Treated-*mdx*). Relative values of differentially expressed metabolites described as fold changes to standard peaks. **(B)** Metabolite concentrations in the skeletal muscles are described for 30–90-day-old mice (P30, P60, and P90). Statistical differences between WT vs. DMD (^*^ *p* < 0.05, ^**^ *p* < 0.01, ^***^ *p <* 0.001, and ^****^ *p <* 0.0001) and DMD vs. Treated-DMD (^*##*^ *p* < 0.01) are indicated; ns, not significant, two-way ANOVA or multiple *t*-test. n = 3 for each group. Data are presented as the mean ± SD.

To compare the exact metabolite amounts, we focused on the quantification of their areas, as reported by the peaks of the mass spectrum. We found that some metabolites, such as phosphocreatine, homocarnosine, *S*-methylcysteine, guanidinosuccinic acid, and thiamine, were altered in the plasma samples of the 60–90-day-old *mdx* mice compared to those in the WT. Among these metabolites, statistical analysis confirmed that *S*-methylcysteine in the DPSC-treated mice at P90 showed significant differences compared to that in the *mdx* mice (Figure 3A). Analysis of plasma samples from 30 to 90-day-old mice among the three groups showed that the metabolites creatine, inosine monophosphate (IMP), and carnosine accumulated in the early phase of the *mdx* mice at the age of 30–90 days (Figure 3B). Additionally, glucose 6-phosphate (G-6-P), fructose 6-phosphate, and thymidine levels were higher in the *mdx* mice than in WT mice after the age of 60 days. In contrast, fumaric acid was reduced in the *mdx* mice after the age of 60 days than in WT mice. Creatine, IMP, G-6-P, and fumaric acid levels in DPSC-treated *mdx* mice were not significantly different from those in WT mice at 90 days of age. Furthermore, in the quantification data of the skeletal muscle, the metabolites, threonic acid, pantothenic acid, *O*-acetylhomoserine, sphinganine, sphingosine, ethanolamine phosphate, and uric acid accumulated in the *mdx* mice at the age of 60–90 days, whereas homocarnosine levels decreased compared to those in WT mice (Figure 4A). Especially, the elevation of Ans and uric acid in the *mdx* mice after DPSC treatment showed a reduction at P90, indicating significant differences when compared to the untreated *mdx* mice (DMD vs. treated-med, Ans, *p* = 0.008; uric acid, *p* = 0.006). Next, we examined the changes in metabolites in the skeletal muscles of mice at the ages of 30–90 days by comparing the three groups of mice (Figure 4B). Choline, creatine, and putrescine levels in the *mdx* mice increased at 60 and 90 days of age. The metabolites choline and Asn were downregulated in DPSC-treated *mdx* mice (Figures 4A, B). While the levels of carnosine in the *mdx* mice were lower than those in WT mice, the differences were not significant when compared with the levels in WT mice after DPSC treatment. In addition, we found that cis-5,8,11,14,17-eicosapentaenoic acid, nicotinamide, and ornitin were transiently elevated in the skeletal muscles of DPSC-treated mice compared with those in the WT and *mdx* mice (Supplementary Table S4; Supplementary Figure S4).

### 3.5 Characterization of disease-specific modified features and the effects of DPSC treatment on the metabolite pathway determined using ingenuity pathway analysis

To understand the significance of the metabolites that underwent disease-specific changes or that varied after DPSC administration, we carried out “diseases and biological functions” analysis using IPA. To detect early changes, we performed the IPA analysis at P60, immediately following DPSC administration. The results revealed canonical pathways that were altered in the plasma samples of mice in the DPSC-treatment group. A total of 21 pathways with predicted upper or lower activation states were detected when untreated *mdx* mice and DPSC-treated *mdx* mice were compared (Table 3). As predicted by the activation z-score, the top pathways activated in *mdx* mice included “transport of amino acids, mobilization of Ca^2+^, uptake of D-glucose, release of nitric oxide, synthesis of prostaglandin E2”. Hence, the relevant factors were reduced in DPSC-treated mice. The top pathways in the *mdx* mice included “uptake of amino acids, storage or concentration of triacylglycerol, uptake of glutamine family amino acid, and conversion of lipid.” Next, we confirmed the differences in these parameters between WT and *mdx* mice along with variability ratios in the untreated and DPSC-treated *mdx* mice (Table 4). We found that the “transport of L-amino acid” and “uptake of L-amino acid” were enriched in the *mdx* mice compared with those in the WT. In contrast, “conversion of lipid” and “uptake of amino acids” were decreased in *mdx* mice. The pathways that were altered in the untreated *mdx* mice compared with those in the WT were increased by approximately two-fold in *mdx* mice compared to those in the DPSC-treated group, except for the “uptake of L-amino acid.” These results imply that the enriched pathways in the *mdx* mice were downregulated in DPSC-treated *mdx* mice, resembling WT mice closely. In contrast, pathways such as the “uptake of amino acids,” “concentration of triacylglycerol,” “uptake of glutamine family amino acid,” and “conversion of lipid,” which were downregulated in the *mdx* mice compared with those in the WT, were upregulated in the DPSC-treated group, indicating a trend toward similarity with WT mice in the DPSC-treated group.

**TABLE 3:** Disease or function annotation using ingenuity pathway analysis (IPA) The plasma samples derived from 60-day-old (P60) untreated *mdx* mice (DMD) and DPSC-treated *mdx* mice (treated-*mdx*) mice were compared by “Disease or function annotation analysis” using IPA and listed as predicted by the activation z-score (>2.0, or < −2.0).

Disease or function annotation	*p*-value	Predicted activation state	Activation z-score	# Molecules
Transport of alpha-amino acid	1.71E-04	Increased	2.902	12
Mobilization of Ca2^+^	8.78E-06	Increased	2.891	24
Transport of neutral amino acid	9.49E-05	Increased	2.772	9
Transport of L-amino acid	6.44E-04	Increased	2.737	11
Efflux of L-amino acid	1.81E-04	Increased	2.588	10
Stimulation of neurons	7.96E-04	Increased	2.538	11
Excitation of neurons	1.03E-03	Increased	2.526	10
Stimulation of cells	3.09E-04	Increased	2.424	18
Export of molecule	1.81E-08	Increased	2.377	31
Synthesis of prostaglandin	1.96E-04	Increased	2.279	17
Proliferation of pancreatic cells	1.78E-03	Increased	2.219	6
Uptake of D-glucose	6.10E-03	Increased	2.208	15
Release of nitric oxide	3.12E-03	Increased	2.184	13
Synthesis of prostaglandin E2	1.04E-03	Increased	2.095	14
Cell viability of tumor cell lines	8.43E-05	Increased	2.059	23
Uptake of amino acids	8.77E-07	Decreased	−3.172	23
Uptake of L-amino acid	1.29E-06	Decreased	−3.069	20
Storage of triacylglycerol	3.62E-04	Decreased	−2.222	5
Concentration of triacylglycerol	1.17E-05	Decreased	−2.194	21
Uptake of glutamine family amino acid	1.38E-05	Decreased	−2.177	14
Conversion of lipid	7.94E-09	Decreased	−2.08	36

**TABLE 4:** Disease and bio function determined using IPA The plasma samples derived from 60-day-old wild type (WT), untreated *mdx* mice, and DPSC-treated *mdx* (treated-*mdx*) mice were compared by “Disease and bio function” using IPA, and the items were listed according to the ratio of the results of *mdx* vs. treated-*mdx* mice (>2.0, or < −2.0). Corresponding values obtained after a comparison between *mdx* vs. WT were also included.

Disease and bio function	Ratio	
	*mdx* vs. WT	*mdx vs*. Treated *mdx*
Transport of alpha-amino acid	−3.74	2.90
Mobilization of Ca^2+^	0.73	2.89
Transport of neutral amino acid	−1.17	2.77
Transport of L-amino acid	41.29	2.74
Efflux of L-amino acid	−1.78	2.59
Stimulation of neurons	0.90	2.54
Excitation of neurons	1.24	2.53
Stimulation of cells	0.55	2.42
Export of molecule	2.92	2.38
Synthesis of prostaglandin	0.47	2.28
Proliferation of pancreatic cells	1.80	2.22
Uptake of D-glucose	0.00	2.21
Release of nitric oxide	1.40	2.18
Synthesis of prostaglandin E2	0.62	2.10
Cell viability of tumor cell lines	19.32	2.06
Uptake of amino acids	−7.60	−3.17
Uptake of L-amino acid	18.24	−3.07
Storage of triacylglycerol	0.82	−2.22
Concentration of triacylglycerol	−4.20	−2.19
Uptake of glutamine family amino acid	−2.60	−2.18
Conversion of lipid	−50.03	−2.08

**FIGURE 5:**
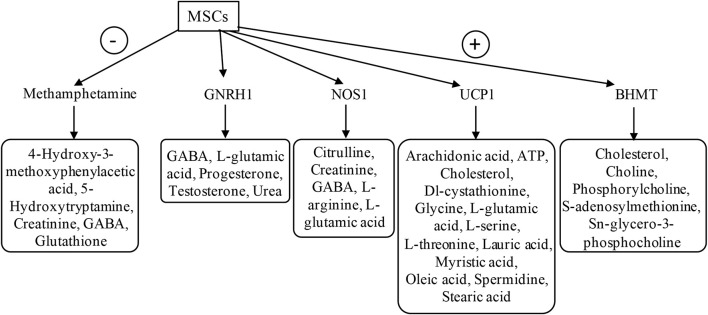
Upstream regulator analysis using IPA The plasma samples derived from 60-day-old untreated *mdx* mice and DPSC-treated *mdx* mice (treated-*mdx*) were compared using “Upstream regulator” analysis by IPA and were listed by activated upstream regulators for both downregulated factors (-; Inhibited, activation z-score; < −2.0), and upregulated factors (+; Activated, activation z-score; >2.0). The target molecules listed based on each upstream factor are surrounded by a square.

IPA was used to identify the upstream regulators that explained the observed changes in the metabolites because upstream regulators were activated early and subsequently contributed to downstream metabolic changes (Figure 5; Supplementary Table S5). This suggests that several upstream regulators are critical for DMD and MSC treatment. The most activated upstream regulators in WT vs. *mdx* mice included disease-specific factors, LEP, NOS3, and 3-nitropropionic acid, while the inhibited state targeted 6–8 molecules such as creatinine, D-sphingosine, glutathione, and L-arginine. LDL and BHMT in the activated state targeted 5–6 molecules such as cholesterol, D-sphingosine, and glutathione.

In the case of DPSC-treated *mdx* mice, the top activated upstream regulators included methamphetamine in an inhibitory state targeting molecules such as 4-hydroxy-3-methoxyphenylacetic acid, 5-hydroxytryptamine, creatinine, GABA, and glutathione as predicted, and upstream regulators BHMT, UCP1, NOS1, and GNRH1 in the activated state, targeting molecules such as creatinine, GABA, L-arginine, L-glutamic acid, oleic acid, phosphorylcholine, and glycero-3-phosphocholine. These results demonstrated that disease-specific or DPSC-treated DMD-specific pathways, including upstream regulators, could be identified using IPA.

## 4 Discussion

We investigated the metabolic signatures of previously identified and newly characterized factors associated with the effects of DPSC treatment on the metabolic status of dystrophic mice. Although a previous study reported metabolic disturbances in DMD using mouse and dog models (Nitahara-Kasahara et al., 2021), this study is the first to report the therapeutic evaluation of cell therapy focused on metabolic abnormalities in DMD. We identified DMD-specific impairments in metabolites and their responses to systemic DPSC treatment. Our results demonstrate that metabolomics-based pathological assessment is expected to help understand the histopathological mechanisms associated with the anti-inflammatory effects of DPSC treatment and can help identify the therapeutic targets and pathways.

Early systemic DPSC administration in *mdx* mice ameliorated the progressive phenotypes and retained milder histological phenotypes (Figure 1). DPSC-treated mice retained motor function, resulting in the long-term improvement of skeletal muscles, as in our previous study (Nitahara-Kasahara et al., 2021). However, the therapeutic mechanisms, target molecules, and environmental changes in the skeletal muscle after systemic administration of DPSCs were unclear. As chronic inflammation is followed by the controlled degeneration and necrosis of muscle fibers, it was considered that the treated mice might have efficient energy production, metabolism, and structural stability, which may be closely related to muscle function.

PCA and heat maps showed that the overall variants of metabolites depended on the time course in *mdx* mice and DPSC-treated *mdx* mice compared to those in WT mice (Figure 2). Quantitative analysis provided a list of metabolites with disease-specific variations, and groups of factors that were further altered by cell therapy were obtained (Tables 1, 2). Furthermore, we focused on the marked differences between normal and disease conditions during the growth process (Figures 3, 4) and suggested that the metabolite variety was affected by DPSC treatment during disease progression.

In dystrophic muscles, disruption of the dystrophin-glycoprotein complex initiates complex pathogenesis, including membrane microrupturing, Ca^2+^-induced proteolytic degradation, and fiber degeneration, followed by chronic inflammation (Allen et al., 2016; Smith, and Barton, 2018; Tidball et al., 2018). In addition to progressive muscle wasting, metabolic abnormalities have been reported previously. For instance, changes in plasma metabolites have been observed in patients with DMD, including alterations in unsaturated fatty acids, carnitine, and lipids. Moreover, metabolites associated with amino acid metabolism, such as elevated levels of Gln and Glu, as well as decreased levels of Val, have been noted compared to healthy controls. (Xu et al., 2023). In an animal model, the metabolites Ala, Met, Gly, and Glu showed different variants in dystrophic *mdx* mice (Lorena et al., 2023) compared to those in the WT. Glu accumulates in GRMD (Kornegay, 2017); however, this alteration may be specific to certain animal models. Our study revealed that DPSC treatments may influence variations in amino acid metabolites, particularly those that are disease-specific and altered in *mdx* mice. This effect is possibly attributed to elevated levels of Asn and Glu (Figure 4A; Supplementary Tables S3, S4).

We found that elevated uric acid and reduced carnosine levels in the skeletal muscle of *mdx* mice showed a trend similar to that of WT mice after DPSC treatment (Figures 4A, B). Similarly, patients with DMD had much higher muscle concentrations of uric acid than healthy individuals, which is associated with purine metabolism (Camina et al., 1995). The hypothesis that DMD involves alterations leading to the blockage of the IMP-purine pathway was supported. Therefore, inhibition of xanthine oxidase is expected to delay the loss of hypoxanthine in the form of uric acid, thus favoring the restoration of nucleotide levels via IMP and guanine (Camina et al., 1995). Considering this pathway, the IMP levels in plasma after the treatment of dystrophic mice with DPSCs were comparable to those in the untreated controls and were not sufficient to restore purine levels in our study (Figure 3B).

Carnosine is a dipeptide that is highly concentrated in the skeletal muscles (Kohen et al., 1988). A previous study showed that significantly decreased carnosine in GRMD could set the stage for eventual muscle damage (e.g., due to lactic acid or oxidative damage). This decline may lead to limited myosin ATPase activity, as highlighted in a previous study (Rayment, 1996), and may contribute to muscle fatigue (Parker, and Ring, 1970). Patients also have significantly lower muscle concentrations of ATP, ADP, GTP, GDP, IMP, S-AMP, hypoxanthine, and guanine (Camina et al., 1995). Our results showed that carnosine levels in the skeletal muscle of DPSC-treated mice were similar to those in WT mice (Figure 4B). We also demonstrated that the locomotor function of *mdx* mice was maintained for a long time after DPSC treatment (Nitahara-Kasahara et al., 2021). These results suggest that energy production and metabolic efficiency may also contribute to the maintenance of muscle function, followed by decreased muscle damage by downregulating inflammation via DPSCs. Future investigations of the relationship between changes in muscle fiber type and metabolic variation may provide clues to explain the mechanisms of the therapeutic effects by DPSC-treatment. Furthermore, higher levels of metabolites involved in glycolysis, including 6-phosphoglycerate, fructose-6-phosphate, and glucose-6-phosphate have been previously reported in *mdx* mice (Xu et al., 2023). Similar results were obtained in this study, and the abnormal metabolism of amino acids, energy, and lipids in DMD was consistent with pathological features, such as recurrent muscle necrosis and regeneration and inflammation, which also partially reflect therapeutic effects.

Choline-containing compounds were approximately three times higher than those in healthy individuals and patients with other myopathies, whereas creatine levels were within the normal range, indicating that abnormal cell membrane function may be correlated with abnormal dystrophin or lack of dystrophin in the brains of patients with DMD (Kato et al., 1997). We found that increased choline in the skeletal muscle of *mdx* mice was similar to that in patients with DMD and was downregulated after DPSC-treatment (Figure 4B). These findings are consistent with the protective cell membrane function in DPSC-treated dystrophic muscle compared with that in the untreated control. Lower levels of circulating CK in the treated mice imply the retention of cellular fragility, albeit temporarily, associated with an improved histological appearance of the skeletal muscle. Therefore, these measurements imply that tissue damage was decreased by DPSC-treatments, reflecting protection against physical damage. In contrast, sphingolipid biosynthesis is known to be upregulated in dystrophic muscles (Laurila et al., 2022), but was not significantly influenced by DPSC treatment.

The IPA pathway is disease-specific and influences various signaling pathways. We found that the main differences between disease and treatment groups included the variation related to increased “transport of amino acid and mobilization of Ca^2+^” and decreased “uptake of amino acids and storage of triacylglycerol” in the analysis of “Diseases or functions annotation” (Table 3). Choline was included as a factor in the “triacylglycerol enrichment or storage” category with a reduced predicted activation state. Carnosine and Glu were also in the “lipid metabolism” Pathway. Creatine and uric acid were in “release of nitric oxide, stimulation of cells, and mobilization of Ca^2+^.” As nitric oxide and Ca^2+^ are known to be involved in the pathogenesis of muscle dystrophy, DPSCs are expected to have the potential to maintain tissue structure. Furthermore, BHMT was identified as an upstream molecule that regulates factors, including choline and its analogs (Figure 5; Supplementary Table S5). There are many upstream factors that regulate Glu; here, we identified UCP1, GNRH1, and NOS1. The dystrophic muscle membrane induces the secondary loss of neuronal nitric oxide synthase. Because nitric oxide is a potent regulator of skeletal muscle metabolism, the loss of NO bioavailability is likely a key contributor to chronic pathology (Timpani et al., 2017). Considering the above findings and our results using IPA based on metabolite variation, we successfully characterized pathways that reflect disease pathology and suggest that treatment may ameliorate disease progression.

There are a few reports on metabolites resulting from MSC administration, for example, MSC-triggered metabolomic alterations in liver-resident immune cells from acute liver injury model mice (Shi et al., 2019), and key metabolic pathways in MSC-mediated immunomodulation for GVHD (Burnham et al., 2020). This is the first report of metabolic improvements following DPSC administration in relation to muscular dystrophy. Whether the supply of DPSCs has a significant impact on the metabolic network and crosstalk with the immune response to alter the disease progression of DMD needs to be further investigated. Metabolite analysis may be useful for understanding the molecular targets and mechanisms underlying cellular therapies.

## Data Availability

The original contributions presented in the study are included in the article/Supplementary Material, further inquiries can be directed to the corresponding authors.
