# Supplementary material for: Characterization of disease-specific alterations in metabolites and effects of mesenchymal stromal cells on dystrophic muscles
Source: Front Cell Dev Biol. 2024 Jun 14;12:1363541. doi: 10.3389/fcell.2024.1363541 (PMC11211584; doi:10.3389/fcell.2024.1363541)
Supplement: Supplementary file 1 [file DataSheet1.docx]

Supplementary Material

# Supplementary Figures and Tables

## Supplementary Figures


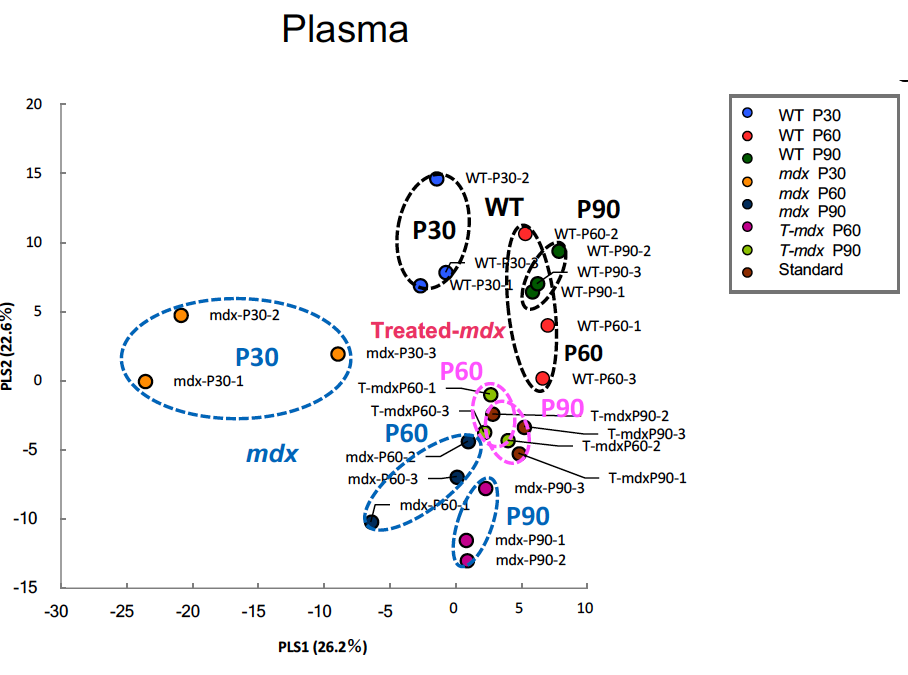


**Supplementary Figure 1.** **Metabolite variation analyzed using the partial least-squares (PLS) method**

Time-series variation in the metabolites analyzed using the partial least-squares method (PLS) are described in three mouse groups; 30-, 60-, and 90-day-old (P30, P60, and P90) C57BL/6 mice (wild type, WT), untreated *mdx* mice (DMD), and DPSC-treated *mdx* mice (Treated-*mdx;* P60, and P90). Three mice per group are encircled by dashed lines.

**
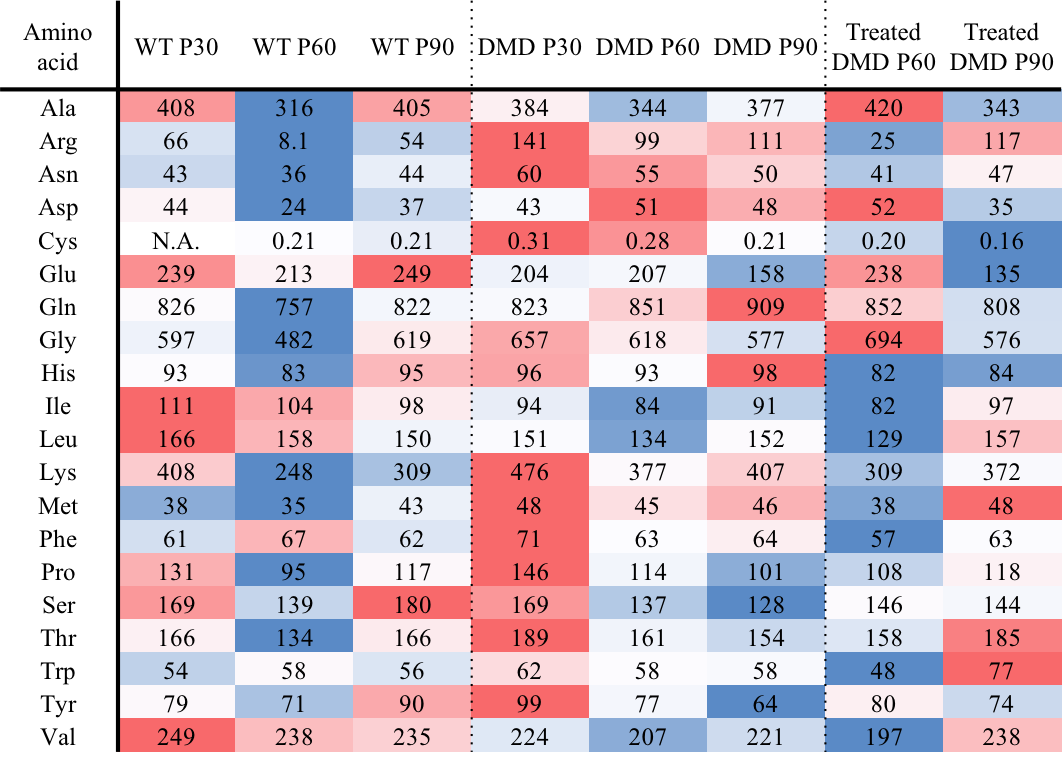

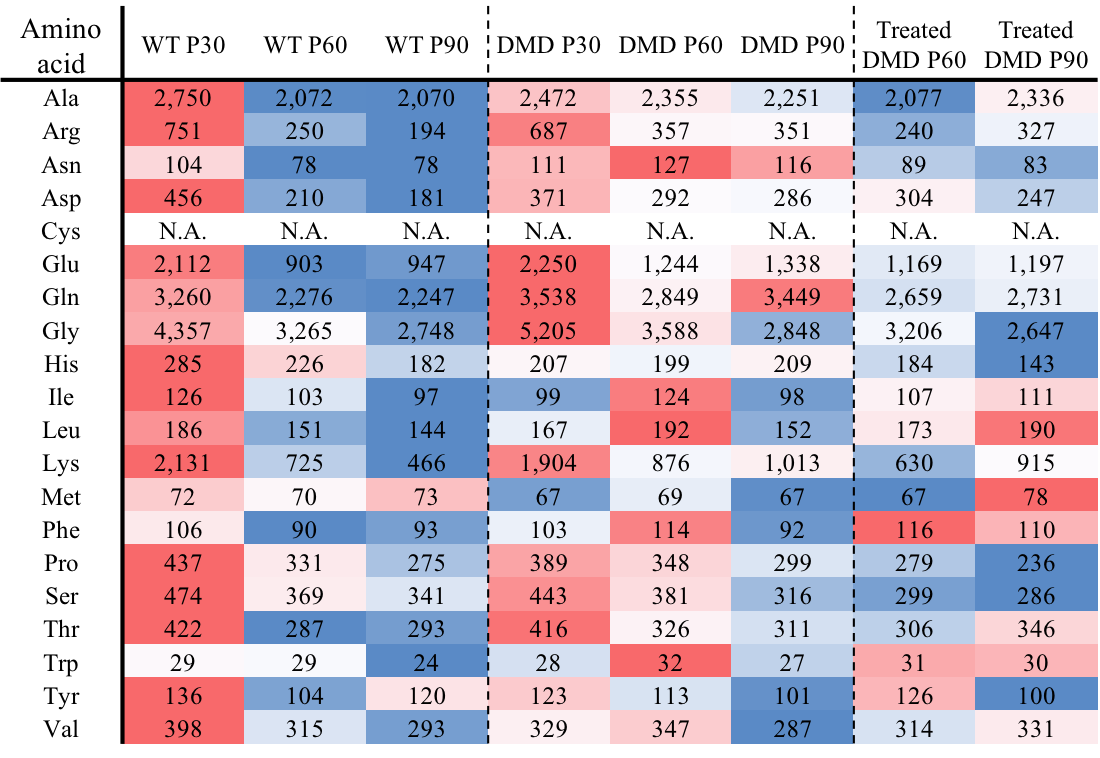
 A B**

Plasma

Skeletal muscle

**C**

Plasma

**
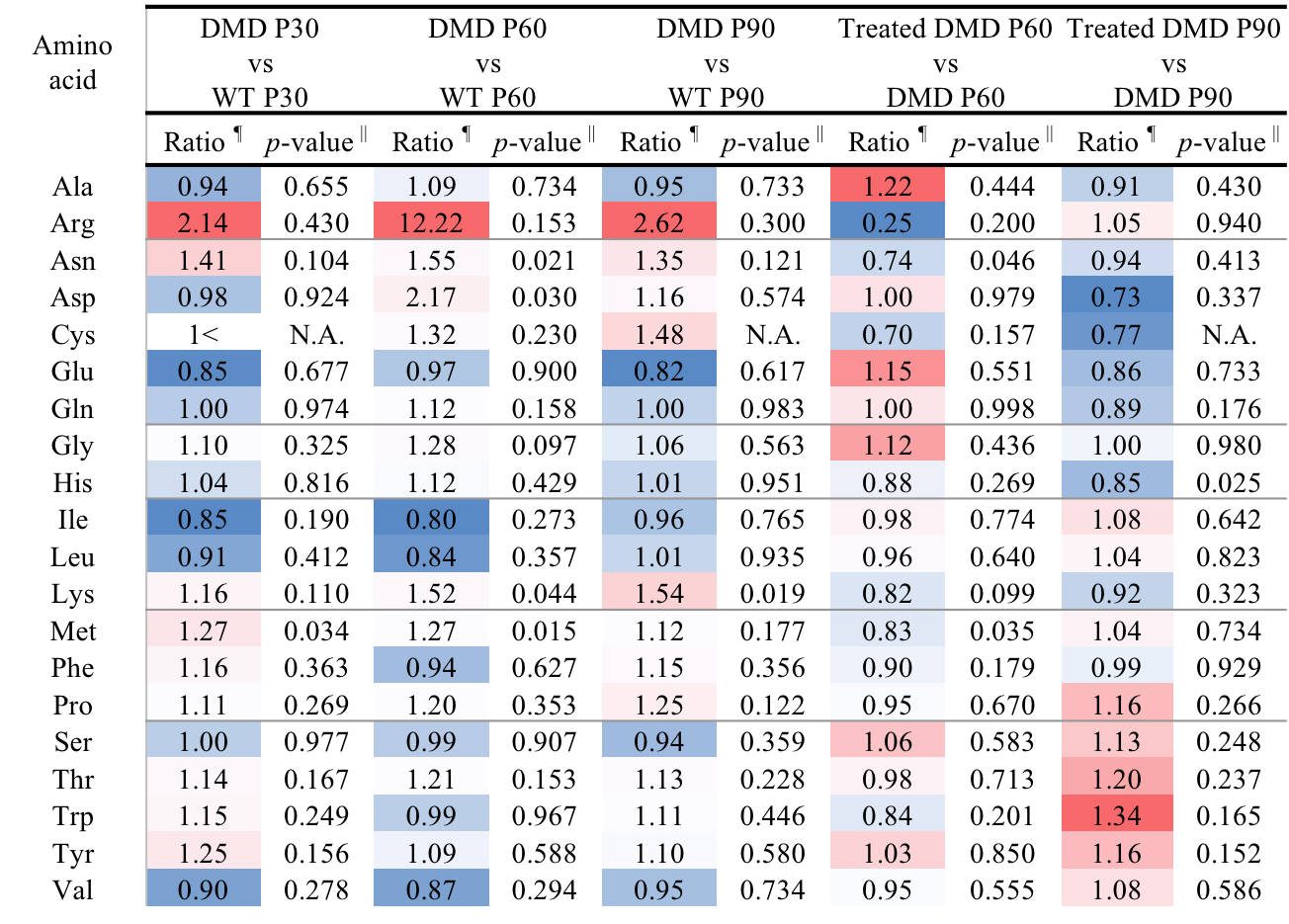
**

**D**

Skeletal muscle

**
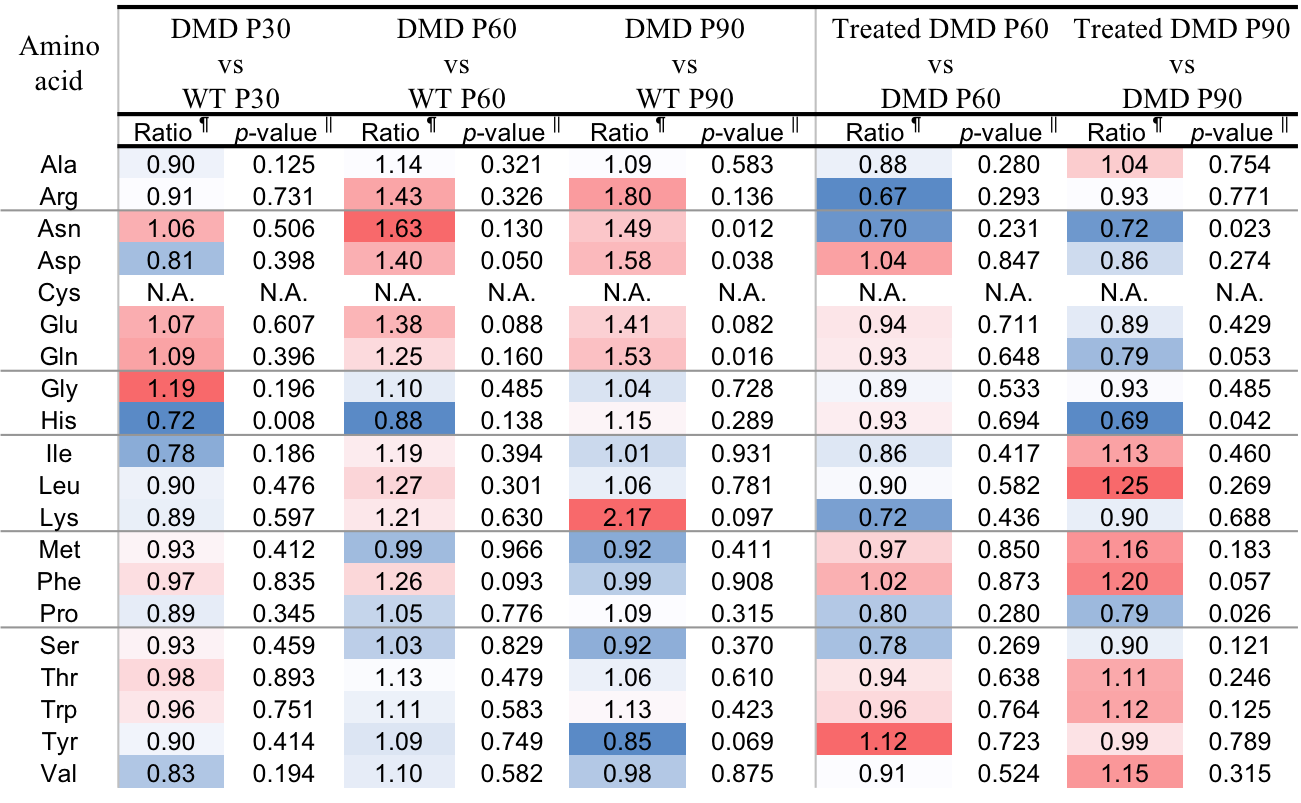
**

**Supplementary Figure 2. Comparative analysis of amino acid metabolites in DMD and DPSC-treated mice**

Quantified amino acid metabolites altered in plasma **(A)** and the tibialis anterior muscles **(B) derived** from 30–90-day-old (P30, P60, and P90) C57BL/6 mice (wild type, WT), untreated *mdx* mice (DMD), and DPSC-treated *mdx* mice (Treated-*mdx*). Relative ratio of differentially expressed metabolites concentration in plasma **(C)** and the TA muscles **(D)** described as fold changes compared to standard peaks. High and low concentration of metabolites are shown on a continuum from red to blue, respectively. Statistical differences are indicated as *p*-values; N.A., Not Available, *t*-test. n = 3 for each group. Data are presented as the mean ± SD.

**Supplementary Figure 3. DPSC-mediated changes in the plasma levels of metabolites**

Plasma levels of metabolites were analyzed from 30–90-day-old mice (P30, P60, and P90). Statistical differences between WT vs. DMD (*^**^P* < 0.01) are indicated; ns, not significant, two-way ANOVA, or multiple *t*-test. n = 3 for each group. Data are presented as the mean ± SD.

**Supplementary Figure 4. DPSC-mediated changes in the levels of metabolites in the skeletal muscles**

The skeletal muscle levels of metabolites concentration were analyzed in 30–90-day-old mice (P30, P60, and P90). Statistical differences between DMD vs. Treated-DMD (*^#^P* < 0.05) groups are indicated; ns, not significant, two-way ANOVA or multiple *t*-test. n = 3 for each group. Data are presented as the mean ± SD.

## Supplementary Tables

**Supplementary Table S1.**

Capillary electrophoresis mass spectrometer (CE-MS) and liquid chromatography-mass spectrometer (LC-MS) analyses were performed using plasma samples derived from 30–90-day-old (P30, P60, and P90) C57BL/6 mice (wild type, WT), untreated *mdx* mice (DMD), and DPSC-treated *mdx* mice (Treated-*mdx*). N = 3 for each group. The table presents the count of peaks corresponding to candidate compound names (both Cation and Anion) derived from the m/z and MT values of substances cataloged in the HMT Metabolite Library. The detection rate was calculated based on the number of detected substances per 451substances.

|  |  |  | |  | |  |  |
| --- | --- | --- | --- | --- | --- | --- | --- |
| Sample name | Group name | CE-MS  Cation Anion | | LC-MS  Positive Negative | | Number of detected substances [/451substances] | Detection rate |
| WT-P30-1 | WT P30 | 153 | 69 | 52 | 74 | 348 | 77.2% |
| WT-P30-3 |  | 171 | 75 | 46 | 58 | 350 | 77.6% |
| WT-P30-4 |  | 163 | 76 | 55 | 42 | 336 | 74.5% |
| WT-P60-1 | WT P60 | 148 | 73 | 45 | 61 | 327 | 72.5% |
| WT-P60-2 |  | 170 | 78 | 45 | 44 | 337 | 74.7% |
| WT-P60-3 |  | 161 | 66 | 54 | 48 | 329 | 72.9% |
| WT-P90-1 | WT P90 | 168 | 75 | 52 | 64 | 359 | 79.6% |
| WT-P90-2 |  | 168 | 76 | 45 | 52 | 341 | 75.6% |
| WT-P90-3 |  | 156 | 76 | 48 | 64 | 344 | 76.3% |
| *mdx*-P30-1 | *mdx* P30 | 158 | 67 | 53 | 61 | 339 | 75.2% |
| *mdx-*P30-2 |  | 165 | 74 | 54 | 60 | 353 | 78.3% |
| *mdx*-P30-3 |  | 161 | 58 | 60 | 70 | 349 | 77.4% |
| *mdx*-P60-1 | *mdx* P60 | 157 | 66 | 55 | 55 | 333 | 73.8% |
| *mdx*-P60-2 |  | 160 | 69 | 51 | 40 | 320 | 71.0% |
| *mdx*-P60-3 |  | 161 | 71 | 55 | 49 | 336 | 74.5% |
| *mdx*-P90-2 | *mdx* P90 | 149 | 58 | 50 | 73 | 330 | 73.2% |
| *mdx*-P90-3 |  | 149 | 62 | 53 | 46 | 310 | 68.7% |
| mdx-P90-4 |  | 155 | 73 | 56 | 46 | 330 | 73.2% |
| T-*mdx* P60-2 | Treated-*mdx* P60 | 168 | 75 | 51 | 52 | 346 | 76.7% |
| T-*mdx* P60-3 |  | 155 | 61 | 54 | 48 | 318 | 70.5% |
| T-*mdx* P60-4 |  | 161 | 71 | 54 | 52 | 338 | 74.9% |
| T-*mdx* P90-1 | Treated-*mdx* P90 | 164 | 68 | 48 | 46 | 326 | 72.3% |
| T-*mdx* P90-2 |  | 153 | 62 | 43 | 53 | 311 | 69.0% |
| T-*mdx* P90-3 |  | 147 | 55 | 44 | 56 | 302 | 67.0% |
| 16Standard-m | 16Standard | 169 | 78 | 53 | 74 | 374 | 82.9% |

**Supplementary Table S2.**

Capillary electrophoresis mass spectrometer (CE-MS) and liquid chromatography mass spectrometer (LC-MS) analyses were performed using the tibialis anterior muscles sample (volume, about 30 mg, each measurement) derived from 30–90-day-old (P30, P60, and P90) C57BL/6 mice (wild type, WT), untreated *mdx* mice (DMD), and DPSC-treated *mdx* mice (Treated-*mdx*). N = 3 for each group. In the table, the number of peaks (Cation and Anion, each) with candidate compound names is listed, which was from the m/z and MT values of the substances registered in the HMT Metabolite Library. Detection rate was calculated based on the number of detected substances
per 571compounds.

| Sample name | (mg) (CE-TOFMS) | (mg) (LC-TOFMS) | Group name | CE-MS  Cation Anion | | LC-MS  Positive Negative | | Number of detected substances [/571substances] | Detection rate |
| --- | --- | --- | --- | --- | --- | --- | --- | --- | --- |
| WT-P30-1 | 36.5 | 32.4 | WT P30 | 250 | 107 | 53 | 28 | 438 | 76.7% |
| WT-P30-3 | 31.7 | 31.5 |  | 245 | 107 | 55 | 25 | 432 | 75.7% |
| WT-P30-4 | 30.5 | 37.3 |  | 243 | 101 | 54 | 26 | 424 | 74.3% |
| WT-P60-1 | 31.4 | 38.5 | WT P60 | 232 | 97 | 56 | 24 | 409 | 71.6% |
| WT-P60-2 | 31.0 | 32.4 |  | 243 | 97 | 57 | 24 | 421 | 73.7% |
| WT-P60-3 | 34.3 | 31.4 |  | 225 | 100 | 56 | 25 | 406 | 71.1% |
| WT-P90-1 | 32.9 | 35.8 | WT P90 | 239 | 98 | 47 | 23 | 407 | 71.3% |
| WT-P90-2 | 30.0 | 35.5 |  | 210 | 90 | 47 | 24 | 371 | 65.0% |
| WT-P90-3 | 30.8 | 35.4 |  | 220 | 94 | 56 | 26 | 396 | 69.4% |
| *mdx*-P30-1 | 32.0 | 30.0 | *mdx* P30 | 249 | 104 | 53 | 23 | 429 | 75.1% |
| *mdx-*P30-2 | 38.0 | 32.4 |  | 271 | 107 | 48 | 26 | 452 | 79.2% |
| *mdx*-P30-3 | 37.1 | 38.5 |  | 270 | 114 | 56 | 33 | 473 | 82.8% |
| *mdx*-P60-1 | 37.2 | 37.0 | *mdx* P60 | 269 | 107 | 49 | 28 | 453 | 79.3% |
| *mdx*-P60-2 | 30.0 | 38.9 |  | 263 | 107 | 52 | 26 | 448 | 78.5% |
| *mdx*-P60-3 | 33.5 | 39.8 |  | 267 | 102 | 56 | 35 | 460 | 80.6% |
| *mdx*-P90-2 | 31.8 | 38.7 | *mdx* P90 | 263 | 104 | 56 | 32 | 455 | 79.7% |
| *mdx*-P90-3 | 34.4 | 32.3 |  | 263 | 111 | 53 | 24 | 451 | 79.0% |
| mdx-P90-4 | 34.0 | 33.0 |  | 253 | 104 | 43 | 28 | 428 | 75.0% |
| T-*mdx* P60-2 | 32.9 | 31.0 | Treated*-mdx* P60 | 251 | 103 | 51 | 28 | 433 | 75.8% |
| T-*mdx* P60-3 | 31.0 | 32.2 |  | 266 | 107 | 47 | 25 | 445 | 77.9% |
| T-*mdx* P60-4 | 30.4 | 34.0 |  | 259 | 106 | 47 | 27 | 439 | 76.9% |
| T-*mdx* P90-1 | 30.8 | 36.3 | Treated*-mdx* P90 | 266 | 102 | 52 | 27 | 447 | 78.3% |
| T-*mdx* P90-2 | 33.0 | 33.0 |  | 265 | 110 | 41 | 25 | 441 | 77.2% |
| T-*mdx* P90-3 | 32.1 | 30.1 |  | 272 | 110 | 41 | 21 | 444 | 77.8% |
| 16Standard-m | 30.0 | 29.7 | 16Standard | 176 | 76 | 46 | 74 | 372 | 65.1% |

**Supplementary Table S3.** **Comparable analysis of metabolites in plasma sample**

For group comparisons, relative area ratios were calculated for each of the peaks in plasma samples. The substances for which names were identified by the Kyoto Encyclopedia of Genes and Genomes (KEGG) and Human Metabolome Technologies data base (HMDB). The ID consists of the initial letter and serial number of the measurement mode (C; cation mode and A; anion mode). The ratios between the age-matched mouse group were calculated, as shown, for untreated *mdx* mice (DMD) or DPSC-treated *mdx* mice (Treated-DMD) and C57BL/6 mice (wild type, WT); DMD vs. WT, and Treated DMD vs. WT or DMD. Data are presented as the mean, and statistical differences are showed as *p*-value (*^*^P* < 0.05, and *^**^P* < 0.01), *t*-test, n = 3 for each group.

| **ID** | **HMT DB ^†^** | | |  | | | | | | | | | | | | | | | | | | | | |
| --- | --- | --- | --- | --- | --- | --- | --- | --- | --- | --- | --- | --- | --- | --- | --- | --- | --- | --- | --- | --- | --- | --- | --- | --- |
|  | **Compound name** | **KEGG ID** | **HMDB ID** | **DMD P30 vs WT P30** | | | **DMD P60 vs WT P60** | | | **DMD P90 vs WT P90** | | | **Treated DMD P60 vs DMD P60** | | | **Treated DMD P60 vs WT P60** | | | **Treated DMD P90 vs DMD P90** | | | **Treated DMD P90 vs WT P90** | | |
|  |  |  |  | **Ratio ^¶^** | ***p*-value ^\|\|^** | | **Ratio ^¶^** | ***p*-value ^\|\|^** | | **Ratio ^¶^** | ***p*-value ^\|\|^** | | **Ratio ^¶^** | ***p*-value ^\|\|^** | | **Ratio ^¶^** | ***p*-value ^\|\|^** | | **Ratio ^¶^** | ***p*-value ^\|\|^** | | **Ratio ^¶^** | ***p*-value ^\|\|^** | |
| **C_0181** | **1-Methyladenosine** | [**C02494**](http://www.genome.jp/dbget-bin/www_bget?cpd:C02494) | [**HMDB03331**](http://www.hmdb.ca/metabolites/HMDB03331) | **1.3** | **0.002** | ****** | **1.2** | **0.136** |  | **1.5** | **0.083** |  | **0.8** | **0.105** |  | **0.9** | **0.641** |  | **0.8** | **0.543** |  | **1.3** | **0.448** |  |
| **C_0115** | **1-Methylhistidine 3-Methylhistidine** | [**No ID C01152**](http://www.genome.jp/dbget-bin/www_bget?cpd:C01152) | [**HMDB00001 HMDB00479**](http://www.hmdb.ca/metabolites/HMDB00001) | **1.1** | **0.468** |  | **1.3** | **0.068** |  | **1.1** | **0.346** |  | **0.9** | **0.186** |  | **1.1** | **0.017** | ***** | **0.9** | **0.378** |  | **1.0** | **0.855** |  |
| **C_0074** | **1-Methylnicotinamide** | [**C02918**](http://www.genome.jp/dbget-bin/www_bget?cpd:C02918) | [**HMDB00699**](http://www.hmdb.ca/metabolites/HMDB00699) | **1.1** | **0.829** |  | **1.4** | **0.173** |  | **1.3** | **0.010** | ***** | **1.2** | **0.644** |  | **1.8** | **0.347** |  | **1.0** | **0.852** |  | **1.3** | **0.318** |  |
| **C_0140** | **11-Aminoundecanoic acid** | [**C19325**](http://www.genome.jp/dbget-bin/www_bget?cpd:C19325) | **No ID** | **1.0** | **0.982** |  | **0.9** | **0.571** |  | **1.0** | **0.928** |  | **0.9** | **0.511** |  | **0.9** | **0.024** | ***** | **1.3** | **0.444** |  | **1.3** | **0.072** |  |
| **N_0050** | **15(S)-HETE-2** | **No ID** | **No ID** | **16** | **0.249** |  | **4.1** | **0.126** |  | **2.4** | **0.063** |  | **1.0** | **0.924** |  | **4.0** | **0.009** | ****** | **0.7** | **0.233** |  | **1.6** | **0.429** |  |
| **C_0159** | **2'-Deoxycytidine** | [**C00881**](http://www.genome.jp/dbget-bin/www_bget?cpd:C00881) | [**HMDB00014**](http://www.hmdb.ca/metabolites/HMDB00014) | **1.3** | **0.035** | ***** | **1.3** | **0.128** |  | **1.4** | **0.003** | ****** | **0.8** | **0.066** |  | **1.1** | **0.672** |  | **0.7** | **0.004** | ****** | **1.0** | **0.482** |  |
| **A_0074** | **2,3-Diphosphoglyceric acid** | [**C01159**](http://www.genome.jp/dbget-bin/www_bget?cpd:C01159) | [**HMDB01294**](http://www.hmdb.ca/metabolites/HMDB01294) | **1.1** | **0.857** |  | **1.1** | **0.740** |  | **0.2** | **0.027** | ***** | **1.5** | **0.391** |  | **1.7** | **0.335** |  | **1.1** | **0.818** |  | **0.2** | **0.027** | ***** |
| **C_0134** | **2,6-Diaminopimelic acid** | [**C00666**](http://www.genome.jp/dbget-bin/www_bget?cpd:C00666) | [**HMDB01370**](http://www.hmdb.ca/metabolites/HMDB01370) | **0.6** | **0.279** |  | **1.2** | **0.207** |  | **0.5** | **0.058** |  | **<1** | **N.A.** |  | **<1** | **N.A.** |  | **0.8** | **0.078** |  | **0.4** | **0.029** | ***** |
| **A_0024** | **2-Hydroxy-4-methylvaleric acid** | [**C03264**](http://www.genome.jp/dbget-bin/www_bget?cpd:C03264) | [**HMDB00624**](http://www.hmdb.ca/metabolites/HMDB00624) | **1.5** | **0.022** | ***** | **1.3** | **N.A.** |  | **1.3** | **0.135** |  | **0.7** | **0.088** |  | **0.9** | **N.A.** |  | **0.9** | **0.547** |  | **1.2** | **0.169** |  |
| **A_0009** | **2-Hydroxybutyric acid** | [**C05984**](http://www.genome.jp/dbget-bin/www_bget?cpd:C05984) | [**HMDB00008**](http://www.hmdb.ca/metabolites/HMDB00008) | **0.7** | **0.058** |  | **0.4** | **0.037** | ***** | **0.6** | **0.026** | ***** | **0.9** | **0.659** |  | **0.4** | **0.043** | ***** | **1.3** | **0.190** |  | **0.8** | **0.078** |  |
| **A_0031** | **2-Hydroxyglutaric acid** | [**C02630,C01087,C03196**](http://www.genome.jp/dbget-bin/www_bget?cpd:C02630) | [**HMDB00606,HMDB00694**](http://www.hmdb.ca/metabolites/HMDB00606) | **0.9** | **0.815** |  | **0.7** | **0.193** |  | **0.6** | **0.093** |  | **1.0** | **0.995** |  | **0.7** | **0.185** |  | **1.5** | **0.002** | ****** | **0.9** | **0.641** |  |
| **A_0007** | **2-Hydroxyisobutyric acid** | **No ID** | [**HMDB00729**](http://www.hmdb.ca/metabolites/HMDB00729) | **1.1** | **0.719** |  | **1.1** | **0.552** |  | **1.0** | **0.487** |  | **1.4** | **0.057** |  | **1.5** | **0.016** | ***** | **1.0** | **0.803** |  | **1.1** | **0.498** |  |
| **P_0032** | **21-Deoxycortisol-2 Corticosterone Cortexolone** | [**No ID C02140 C05488**](http://www.genome.jp/dbget-bin/www_bget?cpd:C02140) | [**No ID HMDB01547 HMDB00015**](http://www.hmdb.ca/metabolites/HMDB01547) | **1.1** | **0.690** |  | **1.2** | **0.391** |  | **1.0** | **0.905** |  | **0.4** | **0.028** | ***** | **0.5** | **0.024** | ***** | **1.3** | **0.233** |  | **1.3** | **0.335** |  |
| **A_0064** | **3-Indoxylsulfuric acid** | **No ID** | [**HMDB00682**](http://www.hmdb.ca/metabolites/HMDB00682) | **1.3** | **0.653** |  | **1.4** | **0.067** |  | **0.8** | **0.357** |  | **1.2** | **0.071** |  | **1.7** | **0.024** | ***** | **1.6** | **0.186** |  | **1.2** | **0.525** |  |
| **A_0021** | **4-Methyl-2-oxovaleric acid 3-Methyl-2-oxovaleric acid** | [**C00233 C00671,C03465**](http://www.genome.jp/dbget-bin/www_bget?cpd:C00233) | [**HMDB00695 HMDB00491**](http://www.hmdb.ca/metabolites/HMDB00695) | **1.0** | **0.912** |  | **1.3** | **0.029** | ***** | **1.4** | **0.435** |  | **0.8** | **0.181** |  | **1.1** | **0.516** |  | **0.7** | **0.465** |  | **1.0** | **0.829** |  |
| **P_0030** | **AC(12:0)-2** | **No ID** | **No ID** | **1.4** | **0.036** | ***** | **0.8** | **N.A.** |  | **1<** | **N.A.** |  | **0.8** | **0.486** |  | **0.6** | **N.A.** |  | **<1** | **N.A.** |  | **N.A.** | **N.A.** |  |
| **P_0041** | **AC(14:1)-1** | **No ID** | **No ID** | **0.8** | **0.565** |  | **1.6** | **0.311** |  | **2.5** | **0.034** | ***** | **0.9** | **0.757** |  | **1.5** | **0.283** |  | **0.2** | **0.021** | ***** | **0.6** | **0.332** |  |
| **P_0039** | **AC(14:2)-2** | **No ID** | **No ID** | **1.7** | **0.014** | ***** | **1.5** | **0.372** |  | **3.2** | **0.064** |  | **0.8** | **0.545** |  | **1.2** | **0.514** |  | **0.3** | **0.072** |  | **1.1** | **0.315** |  |
| **P_0047** | **AC(15:0)-2** | **No ID** | **No ID** | **1.3** | **0.531** |  | **1.1** | **0.421** |  | **1.0** | **0.900** |  | **1.0** | **0.636** |  | **1.2** | **0.288** |  | **0.8** | **0.524** |  | **0.8** | **0.044** | ***** |
| **P_0056** | **AC(16:1)** | **No ID** | **No ID** | **1.0** | **0.940** |  | **1.0** | **0.978** |  | **2.5** | **0.032** | ***** | **0.9** | **0.530** |  | **0.9** | **0.562** |  | **0.4** | **0.020** | ***** | **0.9** | **0.715** |  |
| **P_0052** | **AC(16:2)-1** | **No ID** | **No ID** | **1.5** | **0.043** | ***** | **1.8** | **N.A.** |  | **1.6** | **0.647** |  | **1.7** | **0.296** |  | **3.0** | **N.A.** |  | **0.8** | **0.850** |  | **1.4** | **0.190** |  |
| **P_0063** | **AC(18:0)** | **No ID** | **No ID** | **1.4** | **0.356** |  | **1.4** | **0.397** |  | **2.0** | **0.045** | ***** | **0.9** | **0.675** |  | **1.2** | **0.364** |  | **0.6** | **0.225** |  | **1.2** | **0.664** |  |
| **P_0061** | **AC(18:2)** | **No ID** | **No ID** | **1.1** | **0.685** |  | **1.2** | **0.645** |  | **3.0** | **0.024** | ***** | **1.1** | **0.717** |  | **1.3** | **0.359** |  | **0.3** | **0.017** | ***** | **0.8** | **0.360** |  |
| **P_0067** | **AC(20:1)** | **No ID** | **No ID** | **1.3** | **0.532** |  | **0.9** | **0.710** |  | **2.5** | **0.002** | ****** | **1.6** | **0.023** | ***** | **1.4** | **0.119** |  | **0.5** | **0.011** | ***** | **1.2** | **0.398** |  |
| **C_0020** | **Acetoacetamide** | [**C11106**](http://www.genome.jp/dbget-bin/www_bget?cpd:C11106) | **No ID** | **1.4** | **0.116** |  | **0.9** | **0.654** |  | **1.0** | **N.A.** |  | **1.6** | **0.016** | ***** | **1.5** | **0.076** |  | **1.4** | **N.A.** |  | **1.4** | **N.A.** |  |
| **C_0141** | **ADMA** | [**C03626**](http://www.genome.jp/dbget-bin/www_bget?cpd:C03626) | [**HMDB01539**](http://www.hmdb.ca/metabolites/HMDB01539) | **1.1** | **0.538** |  | **1.5** | **0.043** | ***** | **1.2** | **0.374** |  | **0.8** | **0.028** | ***** | **1.2** | **0.235** |  | **0.9** | **0.675** |  | **1.1** | **0.730** |  |
| **C_0047** | **Anserine_divalent** | [**C01262**](http://www.genome.jp/dbget-bin/www_bget?cpd:C01262) | [**HMDB00194**](http://www.hmdb.ca/metabolites/HMDB00194) | **3.0** | **0.110** |  | **14** | **0.043** | ***** | **8.8** | **0.036** | ***** | **0.7** | **0.304** |  | **10** | **0.039** | ***** | **0.8** | **0.696** |  | **7.3** | **0.181** |  |
| **N_0041** | **Arachidonic acid** | [**C00219**](http://www.genome.jp/dbget-bin/www_bget?cpd:C00219) | [**HMDB01043**](http://www.hmdb.ca/metabolites/HMDB01043) | **28** | **0.196** |  | **7.9** | **0.462** |  | **4.8** | **0.381** |  | **0.6** | **0.732** |  | **4.9** | **0.011** | ***** | **0.3** | **0.418** |  | **1.3** | **0.732** |  |
| **C_0186** | **Argininosuccinic acid** | [**C03406**](http://www.genome.jp/dbget-bin/www_bget?cpd:C03406) | [**HMDB00052**](http://www.hmdb.ca/metabolites/HMDB00052) | **1.4** | **0.605** |  | **3.4** | **0.048** | ***** | **0.3** | **0.256** |  | **0.8** | **0.538** |  | **2.6** | **0.233** |  | **1.3** | **0.572** |  | **0.4** | **0.316** |  |
| **C_0065** | **Asn** | [**C00152,C01905,C16438**](http://www.genome.jp/dbget-bin/www_bget?cpd:C00152) | [**HMDB00168**](http://www.hmdb.ca/metabolites/HMDB00168) | **1.4** | **0.104** |  | **1.5** | **0.021** | ***** | **1.1** | **0.128** |  | **0.7** | **0.046** | ***** | **1.1** | **0.309** |  | **0.9** | **0.413** |  | **1.1** | **0.562** |  |
| **C_0069** | **Asp** | [**C00049,C00402,C16433**](http://www.genome.jp/dbget-bin/www_bget?cpd:C00049) | [**HMDB00191,HMDB06483**](http://www.hmdb.ca/metabolites/HMDB00191) | **1.0** | **0.924** |  | **2.2** | **0.030** | ***** | **1.3** | **0.305** |  | **1.0** | **0.979** |  | **2.2** | **0.051** |  | **0.7** | **0.337** |  | **0.9** | **0.881** |  |
| **C_0040** | **Betaine** | [**C00719**](http://www.genome.jp/dbget-bin/www_bget?cpd:C00719) | [**HMDB00043**](http://www.hmdb.ca/metabolites/HMDB00043) | **1.3** | **0.145** |  | **1.1** | **0.667** |  | **0.8** | **0.145** |  | **0.9** | **0.470** |  | **0.9** | **0.731** |  | **0.9** | **0.340** |  | **0.7** | **0.021** | ***** |
| **C_0161** | **Butyrylcarnitine** | [**C02862**](http://www.genome.jp/dbget-bin/www_bget?cpd:C02862) | [**HMDB02013**](http://www.hmdb.ca/metabolites/HMDB02013) | **0.5** | **0.035** | ***** | **0.6** | **0.053** |  | **0.7** | **0.181** |  | **1.0** | **0.801** |  | **0.5** | **0.060** |  | **0.8** | **0.367** |  | **0.6** | **0.058** |  |
| **P_0045** | **Campesterol** | [**C01789**](http://www.genome.jp/dbget-bin/www_bget?cpd:C01789) | [**HMDB02869**](http://www.hmdb.ca/metabolites/HMDB02869) | **0.9** | **0.634** |  | **0.9** | **0.323** |  | **0.8** | **N.A.** |  | **0.6** | **0.057** |  | **0.5** | **0.006** | ****** | **1.1** | **0.734** |  | **0.9** | **N.A.** |  |
| **C_0158** | **Carnosine** | [**C00386**](http://www.genome.jp/dbget-bin/www_bget?cpd:C00386) | [**HMDB00033**](http://www.hmdb.ca/metabolites/HMDB00033) | **9.8** | **0.012** | ***** | **18** | **0.001** | ****** | **11** | **0.011** | ***** | **1.0** | **0.781** |  | **17** | **0.008** | ****** | **0.9** | **0.857** |  | **9.8** | **0.049** | ***** |
| **C_0027** | **Choline** | [**C00114**](http://www.genome.jp/dbget-bin/www_bget?cpd:C00114) | [**HMDB00097**](http://www.hmdb.ca/metabolites/HMDB00097) | **0.8** | **0.167** |  | **0.7** | **0.034** | ***** | **0.9** | **0.060** |  | **1.0** | **0.966** |  | **0.7** | **0.077** |  | **0.8** | **0.276** |  | **0.7** | **0.160** |  |
| **N_0045** | **cis-11,14-Eicosadienoic acid-2** | **No ID** | **No ID** | **6.6** | **0.158** |  | **1.7** | **0.361** |  | **3.1** | **0.014** | ***** | **0.8** | **0.649** |  | **1.4** | **0.215** |  | **0.3** | **0.037** | ***** | **0.9** | **0.904** |  |
| **N_0051** | ***cis*-4,7,10,13,16,19-Docosahexaenoic acid** | [**C06429**](http://www.genome.jp/dbget-bin/www_bget?cpd:C06429) | [**HMDB02183**](http://www.hmdb.ca/metabolites/HMDB02183) | **18** | **0.190** |  | **6.4** | **0.488** |  | **5.9** | **0.008** | ****** | **0.4** | **0.580** |  | **2.3** | **0.046** | ***** | **0.2** | **0.013** | ***** | **1.4** | **0.636** |  |
| **C_0066** | **Creatine** | [**C00300**](http://www.genome.jp/dbget-bin/www_bget?cpd:C00300) | [**HMDB00064**](http://www.hmdb.ca/metabolites/HMDB00064) | **2.3** | **2.9E-04** | ******* | **4.2** | **5.7E-05** | ******** | **3.7** | **0.003** | ******* | **1.0** | **0.937** |  | **4.0** | **0.001** | ******** | **0.6** | **0.078** |  | **2.4** | **0.077** |  |
| **C_0035** | **Creatinine** | [**C00791**](http://www.genome.jp/dbget-bin/www_bget?cpd:C00791) | [**HMDB00562**](http://www.hmdb.ca/metabolites/HMDB00562) | **1.0** | **0.677** |  | **1.3** | **0.041** | ***** | **1.0** | **0.819** |  | **1.0** | **0.543** |  | **1.2** | **0.129** |  | **1.1** | **0.748** |  | **1.1** | **0.676** |  |
| **C_0155** | **Cystathionine** | [**C00542,C02291**](http://www.genome.jp/dbget-bin/www_bget?cpd:C00542) | [**HMDB00099**](http://www.hmdb.ca/metabolites/HMDB00099) | **0.8** | **0.352** |  | **0.6** | **0.411** |  | **0.6** | **0.032** | ***** | **1.4** | **0.494** |  | **0.8** | **0.709** |  | **1.0** | **0.964** |  | **0.6** | **0.234** |  |
| **C_0165** | **Cystine** | [**C00491,C01420**](http://www.genome.jp/dbget-bin/www_bget?cpd:C00491) | [**HMDB00192**](http://www.hmdb.ca/metabolites/HMDB00192) | **1.5** | **0.015** | ***** | **2.0** | **0.017** | ***** | **2.2** | **0.201** |  | **0.6** | **0.013** | ***** | **1.1** | **0.660** |  | **0.9** | **0.758** |  | **1.9** | **0.133** |  |
| **N_0023** | **Daidzein** | [**C10208**](http://www.genome.jp/dbget-bin/www_bget?cpd:C10208) | [**HMDB03312**](http://www.hmdb.ca/metabolites/HMDB03312) | **1.9** | **0.028** | ***** | **2.8** | **N.A.** |  | **0.8** | **N.A.** |  | **2.8** | **0.210** |  | **7.8** | **N.A.** |  | **0.9** | **N.A.** |  | **0.8** | **0.581** |  |
| **C_0003** | **Ethanolamine** | [**C00189**](http://www.genome.jp/dbget-bin/www_bget?cpd:C00189) | [**HMDB00149**](http://www.hmdb.ca/metabolites/HMDB00149) | **1.2** | **0.340** |  | **1.4** | **0.029** | ***** | **1.1** | **0.706** |  | **0.8** | **0.068** |  | **1.1** | **0.399** |  | **0.8** | **0.205** |  | **0.9** | **0.523** |  |
| **N_0001** | **FA(12:0)** | **No ID** | **No ID** | **2.8** | **0.036** | ***** | **4.3** | **N.A.** |  | **2.0** | **N.A.** |  | **0.7** | **N.A.** |  | **3.2** | **N.A.** |  | **0.2** | **N.A.** |  | **0.4** | **0.400** |  |
| **N_0007** | **FA(14:1)-1-1 FA(14:1)-2-1** | **No ID No ID** | **No ID No ID** | **1.0** | **0.982** |  | **1.6** | **0.230** |  | **3.6** | **0.008** | ****** | **0.7** | **0.388** |  | **1.1** | **0.694** |  | **0.4** | **0.298** |  | **1.4** | **0.760** |  |
| **N_0004** | **FA(14:2)-1** | **No ID** | **No ID** | **0.9** | **N.A.** |  | **2.1** | **0.096** |  | **5.4** | **0.020** | ***** | **1.0** | **0.931** |  | **2.2** | **0.376** |  | **0.5** | **0.491** |  | **2.9** | **0.579** |  |
| **N_0021** | **FA(16:2)-1** | **No ID** | **No ID** | **5.6** | **0.198** |  | **2.2** | **0.440** |  | **3.4** | **0.050** | ***** | **0.6** | **0.517** |  | **1.2** | **0.459** |  | **0.3** | **0.060** |  | **1.1** | **0.897** |  |
| **N_0030** | **FA(17:0)-2 Heptadecanoic acid-2** | **No ID No ID** | **No ID No ID** | **3.9** | **0.209** |  | **1.5** | **0.359** |  | **3.3** | **0.035** | ***** | **0.9** | **0.684** |  | **1.3** | **0.266** |  | **0.3** | **0.055** |  | **1.1** | **0.918** |  |
| **N_0027** | **FA(17:1)** | **No ID** | **No ID** | **6.8** | **0.208** |  | **1.5** | **0.644** |  | **2.4** | **0.041** | ***** | **0.8** | **0.792** |  | **1.2** | **0.517** |  | **0.3** | **0.074** |  | **0.8** | **0.791** |  |
| **N_0037** | **FA(19:1)** | **No ID** | **No ID** | **4.6** | **0.204** |  | **1.1** | **0.889** |  | **2.3** | **0.057** |  | **1.0** | **0.941** |  | **1.0** | **0.908** |  | **0.3** | **0.041** | ***** | **0.7** | **0.676** |  |
| **N_0042** | **FA(20:3)** | **No ID** | **No ID** | **11** | **0.203** |  | **4.4** | **0.432** |  | **4.2** | **0.045** | ***** | **0.6** | **0.643** |  | **2.5** | **0.012** | ***** | **0.09** | **0.039** | ***** | **0.4** | **0.134** |  |
| **N_0056** | **FA(22:4)-2** | **No ID** | **No ID** | **16** | **0.189** |  | **3.6** | **0.327** |  | **5.2** | **0.081** |  | **0.5** | **0.473** |  | **1.8** | **0.005** | ****** | **0.08** | **0.067** |  | **0.4** | **0.107** |  |
| **N_0052** | **FA(22:5)-1** | **No ID** | **No ID** | **16** | **0.193** |  | **4.0** | **0.443** |  | **5.2** | **0.003** | ****** | **0.5** | **0.622** |  | **2.2** | **0.067** |  | **0.3** | **0.024** | ***** | **1.4** | **0.681** |  |
| **N_0054** | **FA(22:5)-3** | **No ID** | **No ID** | **18** | **0.196** |  | **4.4** | **0.298** |  | **4.5** | **3.4E-04** | ******* | **0.6** | **0.567** |  | **2.8** | **0.002** | ****** | **0.3** | **0.050** | ***** | **1.4** | **0.660** |  |
| **N_0065** | **FA(24:5)** | **No ID** | **No ID** | **21** | **0.194** |  | **2.0** | **0.395** |  | **3.9** | **0.017** | ***** | **0.8** | **0.726** |  | **1.6** | **0.163** |  | **0.4** | **0.096** |  | **1.5** | **0.639** |  |
| **A_0071** | **Fructose 6-phosphate** | [**C05345,C00085**](http://www.genome.jp/dbget-bin/www_bget?cpd:C05345) | [**HMDB00124**](http://www.hmdb.ca/metabolites/HMDB00124) | **1.3** | **0.340** |  | **2.7** | **0.001** | ****** | **2.3** | **0.099** |  | **0.9** | **0.852** |  | **2.4** | **0.019** | ***** | **0.7** | **0.384** |  | **1.6** | **0.558** |  |
| **A_0012** | **Fumaric acid** | [**C00122**](http://www.genome.jp/dbget-bin/www_bget?cpd:C00122) | [**HMDB00134**](http://www.hmdb.ca/metabolites/HMDB00134) | **1.0** | **0.768** |  | **1.0** | **0.865** |  | **0.4** | **0.005** | ****** | **1.1** | **0.545** |  | **1.1** | **0.457** |  | **1.6** | **0.136** |  | **0.7** | **0.113** |  |
| **A_0099** | **GDP** | [**C00035**](http://www.genome.jp/dbget-bin/www_bget?cpd:C00035) | [**HMDB01201**](http://www.hmdb.ca/metabolites/HMDB01201) | **1.0** | **0.914** |  | **0.9** | **0.775** |  | **0.7** | **0.022** | ***** | **1.4** | **0.470** |  | **1.3** | **0.568** |  | **0.8** | **0.437** |  | **0.6** | **0.133** |  |
| **A_0073** | **Glucose 1-phosphate** | [**C00103**](http://www.genome.jp/dbget-bin/www_bget?cpd:C00103) | [**HMDB01586**](http://www.hmdb.ca/metabolites/HMDB01586) | **0.3** | **0.015** | ***** | **1.3** | **0.645** |  | **1.1** | **0.898** |  | **1.4** | **0.684** |  | **1.7** | **0.516** |  | **1.4** | **0.785** |  | **1.5** | **0.752** |  |
| **A_0072** | **Glucose 6-phosphate** | [**C00668,C01172,C00092**](http://www.genome.jp/dbget-bin/www_bget?cpd:C00668) | [**HMDB01401**](http://www.hmdb.ca/metabolites/HMDB01401) | **1.2** | **0.631** |  | **2.8** | **0.001** | ****** | **2.9** | **0.030** | ***** | **0.8** | **0.212** |  | **2.2** | **0.043** | ***** | **0.8** | **0.563** |  | **2.3** | **0.1175** |  |
| **A_0001** | **Glyoxylic acid** | [**C00048**](http://www.genome.jp/dbget-bin/www_bget?cpd:C00048) | [**HMDB00119**](http://www.hmdb.ca/metabolites/HMDB00119) | **2.1** | **0.018** | ***** | **2.1** | **N.A.** |  | **0.9** | **0.511** |  | **0.8** | **0.533** |  | **1.7** | **N.A.** |  | **2.8** | **0.117** |  | **2.4** | **0.128** |  |
| **C_0119** | **Guanidinosuccinic acid** | [**C03139**](http://www.genome.jp/dbget-bin/www_bget?cpd:C03139) | [**HMDB03157**](http://www.hmdb.ca/metabolites/HMDB03157) | **0.4** | **0.134** |  | **0.5** | **0.004** | ****** | **0.6** | **0.427** |  | **2.4** | **0.349** |  | **1.1** | **0.895** |  | **0.6** | **0.475** |  | **0.4** | **0.233** |  |
| **C_0038** | **Guanidoacetic acid** | [**C00581**](http://www.genome.jp/dbget-bin/www_bget?cpd:C00581) | [**HMDB00128**](http://www.hmdb.ca/metabolites/HMDB00128) | **1.6** | **0.109** |  | **1.7** | **0.026** | ***** | **1.1** | **0.309** |  | **0.8** | **0.099** |  | **1.3** | **0.147** |  | **0.9** | **0.754** |  | **1.1** | **0.763** |  |
| **C_0102** | **His** | [**C00135,C00768,C06419**](http://www.genome.jp/dbget-bin/www_bget?cpd:C00135) | [**HMDB00177**](http://www.hmdb.ca/metabolites/HMDB00177) | **1.0** | **0.816** |  | **1.1** | **0.429** |  | **1.0** | **0.493** |  | **0.9** | **0.269** |  | **1.0** | **0.927** |  | **0.9** | **0.025** | ***** | **0.9** | **0.032** | ***** |
| **C_0033** | **Histamine** | [**C00388**](http://www.genome.jp/dbget-bin/www_bget?cpd:C00388) | [**HMDB00870**](http://www.hmdb.ca/metabolites/HMDB00870) | **0.7** | **0.293** |  | **1.0** | **0.921** |  | **0.7** | **0.009** | ****** | **1.4** | **0.207** |  | **1.4** | **0.221** |  | **1.0** | **0.961** |  | **0.6** | **0.378** |  |
| **C_0166** | **Homocarnosine** | [**C00884**](http://www.genome.jp/dbget-bin/www_bget?cpd:C00884) | [**HMDB00745**](http://www.hmdb.ca/metabolites/HMDB00745) | **3.1** | **0.100** |  | **15** | **0.048** | ***** | **9.0** | **0.043** | ***** | **0.7** | **0.341** |  | **11** | **0.042** | ***** | **0.9** | **0.785** |  | **7.8** | **0.197** |  |
| **C_0060** | **Hydroxyproline** | [**C01157**](http://www.genome.jp/dbget-bin/www_bget?cpd:C01157) | [**HMDB00725**](http://www.hmdb.ca/metabolites/HMDB00725) | **1.3** | **0.146** |  | **1.8** | **0.010** | ***** | **1.3** | **0.100** |  | **0.9** | **0.343** |  | **1.5** | **0.066** |  | **1.0** | **0.990** |  | **1.3** | **0.143** |  |
| **C_0030** | **Hypotaurine** | [**C00519**](http://www.genome.jp/dbget-bin/www_bget?cpd:C00519) | [**HMDB00965**](http://www.hmdb.ca/metabolites/HMDB00965) | **1.2** | **0.575** |  | **1.7** | **0.157** |  | **0.5** | **0.003** | ****** | **0.7** | **0.350** |  | **1.3** | **0.417** |  | **0.7** | **0.435** |  | **0.4** | **0.028** | ***** |
| **A_0089** | **IMP** | [**C00130**](http://www.genome.jp/dbget-bin/www_bget?cpd:C00130) | [**HMDB00175**](http://www.hmdb.ca/metabolites/HMDB00175) | **1.5** | **0.014** | ***** | **4.0** | **0.004** | ****** | **2.9** | **0.019** | ***** | **1.1** | **0.594** |  | **3.8** | **0.001** | ****** | **0.5** | **0.232** |  | **1.6** | **0.595** |  |
| **A_0041** | **Isovalerylalanine-1 N-Acetylleucine-1** | **No ID No ID** | **No ID No ID** | **<1** | **N.A.** |  | **0.4** | **0.004** | ****** | **1.3** | **0.317** |  | **1.3** | **0.262** |  | **0.5** | **0.008** | ****** | **0.7** | **0.210** |  | **0.9** | **0.595** |  |
| **A_0059** | **Lauric acid** | [**C02679**](http://www.genome.jp/dbget-bin/www_bget?cpd:C02679) | [**HMDB00638**](http://www.hmdb.ca/metabolites/HMDB00638) | **1.2** | **0.099** |  | **0.9** | **0.551** |  | **1.1** | **0.464** |  | **0.9** | **0.225** |  | **0.8** | **0.131** |  | **0.9** | **0.035** | ***** | **1.0** | **0.845** |  |
| **C_0200** | **Leupeptin** | [**C01591**](http://www.genome.jp/dbget-bin/www_bget?cpd:C01591) | **No ID** | **1.0** | **0.932** |  | **2.1** | **0.168** |  | **0.5** | **0.001** | ****** | **0.8** | **0.547** |  | **1.7** | **0.109** |  | **2.2** | **0.058** |  | **1.2** | **0.379** |  |
| **N_0033** | **Linoleic acid** | [**C01595**](http://www.genome.jp/dbget-bin/www_bget?cpd:C01595) | [**HMDB00673**](http://www.hmdb.ca/metabolites/HMDB00673) | **8.6** | **0.200** |  | **2.7** | **0.540** |  | **2.7** | **0.047** | ***** | **0.6** | **0.667** |  | **1.6** | **0.195** |  | **0.3** | **0.070** |  | **0.9** | **0.857** |  |
| **P_0023** | **Linoleyl ethanolamide** | **No ID** | [**HMDB12252**](http://www.hmdb.ca/metabolites/HMDB12252) | **0.8** | **0.581** |  | **1.4** | **0.125** |  | **1.2** | **0.123** |  | **0.9** | **0.832** |  | **1.3** | **0.389** |  | **0.8** | **0.028** | ***** | **1.0** | **0.828** |  |
| **C_0089** | **Lys** | [**C00047,C00739,C16440**](http://www.genome.jp/dbget-bin/www_bget?cpd:C00047) | [**HMDB00182,HMDB03405**](http://www.hmdb.ca/metabolites/HMDB00182) | **1.2** | **0.110** |  | **1.5** | **0.044** | ***** | **1.3** | **0.087** |  | **0.8** | **0.099** |  | **1.2** | **0.227** |  | **0.9** | **0.323** |  | **1.2** | **0.190** |  |
| **A_0025** | **Malic acid** | [**C00149,C00497,C00711**](http://www.genome.jp/dbget-bin/www_bget?cpd:C00149) | [**HMDB00156,HMDB00744**](http://www.hmdb.ca/metabolites/HMDB00156) | **1.0** | **0.710** |  | **1.0** | **0.886** |  | **0.5** | **0.030** | ***** | **1.1** | **0.626** |  | **1.2** | **0.463** |  | **1.6** | **0.168** |  | **0.8** | **0.270** |  |
| **C_0097** | **Met** | [**C00073,C00855,C01733**](http://www.genome.jp/dbget-bin/www_bget?cpd:C00073) | [**HMDB00696**](http://www.hmdb.ca/metabolites/HMDB00696) | **1.3** | **0.034** | ***** | **1.3** | **0.015** | ***** | **1.1** | **0.390** |  | **0.8** | **0.035** | ***** | **1.1** | **0.298** |  | **1.0** | **0.734** |  | **1.1** | **0.440** |  |
| **C_0026** | ***N*,*N*-Dimethylglycine** | [**C01026**](http://www.genome.jp/dbget-bin/www_bget?cpd:C01026) | [**HMDB00092**](http://www.hmdb.ca/metabolites/HMDB00092) | **1.2** | **0.032** | ***** | **0.9** | **0.262** |  | **0.9** | **0.624** |  | **0.9** | **0.172** |  | **0.8** | **0.085** |  | **0.9** | **0.425** |  | **0.8** | **0.331** |  |
| **A_0015** | ***N*-Acetylglycine** | **No ID** | [**HMDB00532**](http://www.hmdb.ca/metabolites/HMDB00532) | **0.8** | **0.325** |  | **0.7** | **0.083** |  | **1.3** | **0.289** |  | **0.8** | **0.159** |  | **0.6** | **0.033** | ***** | **0.7** | **0.277** |  | **1.0** | **0.847** |  |
| **C_0091** | ***N*-Acetylserine** | **No ID** | [**HMDB02931**](http://www.hmdb.ca/metabolites/HMDB02931) | **1.0** | **0.608** |  | **0.9** | **0.620** |  | **0.7** | **5.2E-04** | ******* | **1.0** | **0.849** |  | **0.9** | **0.562** |  | **1.1** | **0.568** |  | **0.8** | **0.125** |  |
| **C_0021** | ***N*-Methylputrescine** | [**C02723**](http://www.genome.jp/dbget-bin/www_bget?cpd:C02723) | [**HMDB03661**](http://www.hmdb.ca/metabolites/HMDB03661) | **1.0** | **0.955** |  | **3.0** | **0.203** |  | **0.7** | **0.144** |  | **0.7** | **0.438** |  | **2.0** | **0.020** | ***** | **1.4** | **0.461** |  | **0.9** | **0.811** |  |
| **C_0135** | ***N*-Methylserotonin** | [**C06212**](http://www.genome.jp/dbget-bin/www_bget?cpd:C06212) | [**HMDB04369**](http://www.hmdb.ca/metabolites/HMDB04369) | **<1** | **N.A.** |  | **<1** | **N.A.** |  | **<1** | **N.A.** |  | **N.A.** | **N.A.** |  | **<1** | **N.A.** |  | **N.A.** | **N.A.** |  | **<1** | **N.A.** |  |
| **C_0101** | ***N*^1^-Methyl-4-pyridone-5-carboxamide** | [**C05843**](http://www.genome.jp/dbget-bin/www_bget?cpd:C05843) | [**HMDB04194**](http://www.hmdb.ca/metabolites/HMDB04194) | **1.1** | **0.565** |  | **1.6** | **0.046** | ***** | **2.0** | **0.006** | ****** | **0.8** | **0.430** |  | **1.3** | **0.529** |  | **0.8** | **0.316** |  | **1.5** | **0.249** |  |
| **C_0117** | ***N*^5^-Ethylglutamine** | [**C01047**](http://www.genome.jp/dbget-bin/www_bget?cpd:C01047) | **No ID** | **1.0** | **0.930** |  | **1.5** | **0.009** | ****** | **1.1** | **0.616** |  | **0.9** | **0.191** |  | **1.3** | **0.070** |  | **0.7** | **0.012** | ***** | **0.8** | **0.372** |  |
| **C_0131** | ***N*^6^,*N*^6^,*N*^6^-Trimethyllysine** | [**C03793**](http://www.genome.jp/dbget-bin/www_bget?cpd:C03793) | [**HMDB01325**](http://www.hmdb.ca/metabolites/HMDB01325) | **1.1** | **0.889** |  | **1.4** | **0.293** |  | **0.6** | **0.044** | ***** | **1.0** | **0.912** |  | **1.5** | **0.342** |  | **0.9** | **0.653** |  | **0.5** | **0.031** | ***** |
| **C_0106** | ***O*-Acetylhomoserine 2-Aminoadipic acid** | [**C01077 C00956**](http://www.genome.jp/dbget-bin/www_bget?cpd:C01077) | [**No ID HMDB00510**](http://www.hmdb.ca/metabolites/HMDB00510) | **0.9** | **0.757** |  | **0.9** | **0.644** |  | **0.7** | **0.113** |  | **1.1** | **0.402** |  | **1.0** | **0.920** |  | **1.0** | **0.809** |  | **0.7** | **0.021** | ***** |
| **N_0034** | **Oleic acid** | [**C00712**](http://www.genome.jp/dbget-bin/www_bget?cpd:C00712) | [**HMDB00207**](http://www.hmdb.ca/metabolites/HMDB00207) | **7.3** | **0.208** |  | **1.3** | **0.723** |  | **2.1** | **0.120** |  | **0.7** | **0.606** |  | **0.9** | **0.636** |  | **0.2** | **0.050** | ***** | **0.5** | **0.300** |  |
| **P_0024** | **Oleoyl ethanolamine** | **No ID** | **No ID** | **1.1** | **0.497** |  | **1.4** | **0.045** | ***** | **1.4** | **0.303** |  | **0.8** | **0.151** |  | **1.1** | **0.626** |  | **0.5** | **0.031** | ***** | **0.7** | **0.374** |  |
| **N_0025** | **Palmitic acid** | [**C00249**](http://www.genome.jp/dbget-bin/www_bget?cpd:C00249) | [**HMDB00220**](http://www.hmdb.ca/metabolites/HMDB00220) | **2.4** | **0.268** |  | **1.1** | **0.786** |  | **2.0** | **0.066** |  | **0.8** | **0.646** |  | **0.9** | **0.722** |  | **0.2** | **0.020** | ***** | **0.3** | **0.156** |  |
| **P_0015** | **Palmitoylethanolamide** | **No ID** | [**HMDB02100**](http://www.hmdb.ca/metabolites/HMDB02100) | **1.7** | **0.132** |  | **2.3** | **0.013** | ***** | **2.3** | **0.100** |  | **0.7** | **0.176** |  | **1.6** | **0.245** |  | **0.5** | **0.042** | ***** | **1.1** | **0.781** |  |
| **A_0065** | **Pantothenic acid** | [**C00864**](http://www.genome.jp/dbget-bin/www_bget?cpd:C00864) | [**HMDB00210**](http://www.hmdb.ca/metabolites/HMDB00210) | **1.1** | **0.691** |  | **1.4** | **0.029** | ***** | **1.1** | **0.680** |  | **1.0** | **0.857** |  | **1.4** | **0.033** | ***** | **1.1** | **0.260** |  | **1.2** | **0.257** |  |
| **A_0063** | **Phosphocreatine** | [**C02305**](http://www.genome.jp/dbget-bin/www_bget?cpd:C02305) | [**HMDB01511**](http://www.hmdb.ca/metabolites/HMDB01511) | **4.0** | **0.004** | ****** | **4.4** | **0.026** | ***** | **4.3** | **0.015** | ***** | **0.8** | **0.378** |  | **3.5** | **0.055** |  | **1.0** | **0.948** |  | **4.3** | **0.044** | ***** |
| **C_0012** | **Putrescine** | [**C00134**](http://www.genome.jp/dbget-bin/www_bget?cpd:C00134) | [**HMDB01414**](http://www.hmdb.ca/metabolites/HMDB01414) | **1.2** | **0.348** |  | **1.6** | **0.276** |  | **1.0** | **0.885** |  | **0.7** | **0.006** | ****** | **1.1** | **0.776** |  | **1.1** | **0.547** |  | **1.2** | **0.517** |  |
| **C_0114** | **Pyridoxal** | [**C00250**](http://www.genome.jp/dbget-bin/www_bget?cpd:C00250) | [**HMDB01545**](http://www.hmdb.ca/metabolites/HMDB01545) | **1.5** | **0.053** |  | **1.1** | **0.729** |  | **1.3** | **0.314** |  | **0.8** | **0.046** | ***** | **0.9** | **0.581** |  | **0.7** | **0.187** |  | **0.9** | **0.591** |  |
| **A_0003** | **Pyruvic acid** | [**C00022**](http://www.genome.jp/dbget-bin/www_bget?cpd:C00022) | [**HMDB00243**](http://www.hmdb.ca/metabolites/HMDB00243) | **1.2** | **0.708** |  | **1.7** | **0.046** | ***** | **1.0** | **0.926** |  | **0.8** | **0.489** |  | **1.4** | **0.412** |  | **0.8** | **0.295** |  | **0.8** | **0.230** |  |
| **C_0070** | ***S*-Methylcysteine** | **No ID** | [**HMDB02108**](http://www.hmdb.ca/metabolites/HMDB02108) | **1.2** | **0.241** |  | **1.2** | **0.206** |  | **1.5** | **0.002** | ****** | **1.2** | **0.048** | ***** | **1.2** | **0.168** |  | **0.8** | **0.043** | ***** | **1.2** | **0.262** |  |
| **C_0142** | **SDMA** | **No ID** | [**HMDB03334**](http://www.hmdb.ca/metabolites/HMDB03334) | **1.2** | **0.207** |  | **1.6** | **0.052** |  | **1.4** | **0.017** | ***** | **0.8** | **0.128** |  | **1.3** | **0.045** | ***** | **0.9** | **0.748** |  | **1.4** | **0.257** |  |
| **C_0028** | **Ser** | [**C00065,C00716,C00740**](http://www.genome.jp/dbget-bin/www_bget?cpd:C00065) | [**HMDB00187,HMDB03406**](http://www.hmdb.ca/metabolites/HMDB00187) | **1.0** | **0.977** |  | **1.0** | **0.907** |  | **0.7** | **0.017** | ***** | **1.1** | **0.583** |  | **1.0** | **0.739** |  | **1.1** | **0.248** |  | **0.8** | **0.056** |  |
| **P_0055** | **Sitosterol** | [**C01753**](http://www.genome.jp/dbget-bin/www_bget?cpd:C01753) | [**HMDB00852**](http://www.hmdb.ca/metabolites/HMDB00852) | **1.1** | **0.895** |  | **1.0** | **0.920** |  | **1.0** | **0.983** |  | **0.6** | **0.035** | ***** | **0.6** | **0.026** | ***** | **1.0** | **0.824** |  | **0.9** | **0.836** |  |
| **C_0084** | **Stachydrine** | [**C10172**](http://www.genome.jp/dbget-bin/www_bget?cpd:C10172) | [**HMDB04827**](http://www.hmdb.ca/metabolites/HMDB04827) | **1.2** | **0.039** | ***** | **1.1** | **0.626** |  | **0.8** | **0.278** |  | **0.9** | **0.162** |  | **1.0** | **0.972** |  | **1.2** | **0.327** |  | **0.9** | **0.671** |  |
| **C_0139** | **Tacrine** | [**C01453**](http://www.genome.jp/dbget-bin/www_bget?cpd:C01453) | **No ID** | **1.1** | **0.750** |  | **1.4** | **0.041** | ***** | **0.8** | **0.021** | ***** | **0.9** | **0.306** |  | **1.3** | **0.077** |  | **1.3** | **0.370** |  | **1.1** | **0.745** |  |
| **C_0051** | **Taurine** | [**C00245**](http://www.genome.jp/dbget-bin/www_bget?cpd:C00245) | [**HMDB00251**](http://www.hmdb.ca/metabolites/HMDB00251) | **1.1** | **0.633** |  | **2.0** | **0.003** | ****** | **2.0** | **0.020** | ***** | **0.9** | **0.468** |  | **1.8** | **0.059** |  | **0.8** | **0.349** |  | **1.6** | **0.272** |  |
| **C_0177** | **Thiamine** | [**C00378**](http://www.genome.jp/dbget-bin/www_bget?cpd:C00378) | [**HMDB00235**](http://www.hmdb.ca/metabolites/HMDB00235) | **0.8** | **0.112** |  | **1.1** | **0.873** |  | **0.6** | **0.012** | ***** | **1.0** | **0.798** |  | **1.0** | **0.926** |  | **1.3** | **0.128** |  | **0.7** | **0.184** |  |
| **C_0090** | ***threo*-β-Methylaspartic acid** | [**C03618**](http://www.genome.jp/dbget-bin/www_bget?cpd:C03618) | **No ID** | **0.5** | **0.004** | ****** | **0.4** | **0.066** |  | **0.5** | **0.223** |  | **1.1** | **0.673** |  | **0.4** | **0.071** |  | **0.8** | **0.490** |  | **0.4** | **0.181** |  |
| **C_0167** | **Thymidine** | [**C00214**](http://www.genome.jp/dbget-bin/www_bget?cpd:C00214) | [**HMDB00273**](http://www.hmdb.ca/metabolites/HMDB00273) | **0.9** | **0.538** |  | **1.4** | **0.139** |  | **1.4** | **0.016** | ***** | **0.9** | **0.804** |  | **1.3** | **0.320** |  | **0.8** | **0.417** |  | **1.1** | **0.659** |  |
| **C_0098** | **Triethanolamine** | [**C06771**](http://www.genome.jp/dbget-bin/www_bget?cpd:C06771) | **No ID** | **0.5** | **0.223** |  | **1.2** | **N.A.** |  | **<1** | **N.A.** |  | **1.1** | **0.795** |  | **1.2** | **N.A.** |  | **1<** | **N.A.** |  | **0.6** | **0.028** | ***** |
| **C_0075** | **Trigonelline** | [**C01004**](http://www.genome.jp/dbget-bin/www_bget?cpd:C01004) | [**HMDB00875**](http://www.hmdb.ca/metabolites/HMDB00875) | **1.3** | **0.163** |  | **0.8** | **0.552** |  | **0.4** | **0.022** | ***** | **1.2** | **0.207** |  | **1.0** | **0.985** |  | **2.0** | **0.118** |  | **0.8** | **0.412** |  |
| **C_0034** | **Uracil** | [**C00106**](http://www.genome.jp/dbget-bin/www_bget?cpd:C00106) | [**HMDB00300**](http://www.hmdb.ca/metabolites/HMDB00300) | **0.8** | **0.050** | ***** | **0.7** | **0.286** |  | **0.7** | **0.064** |  | **0.9** | **0.491** |  | **0.6** | **0.182** |  | **0.9** | **0.674** |  | **0.7** | **0.115** |  |
| **A_0067** | **XA0027** | **No ID** | **No ID** | **1.6** | **0.219** |  | **0.9** | **0.837** |  | **1.9** | **8.2E-04** | ******* | **0.7** | **0.416** |  | **0.7** | **0.165** |  | **0.4** | **0.005** | ****** | **0.9** | **0.385** |  |
| **C_0116** | **XC0040** | **No ID** | **No ID** | **0.8** | **0.477** |  | **0.7** | **0.228** |  | **0.7** | **0.016** | ***** | **1.2** | **0.078** |  | **0.8** | **0.421** |  | **1.1** | **0.503** |  | **0.7** | **0.067** |  |
| **C_0014** | **β-Ala** | [**C00099**](http://www.genome.jp/dbget-bin/www_bget?cpd:C00099) | [**HMDB00056**](http://www.hmdb.ca/metabolites/HMDB00056) | **0.8** | **0.667** |  | **0.7** | **0.458** |  | **0.6** | **0.046** | ***** | **1.4** | **0.515** |  | **1.0** | **0.941** |  | **1.0** | **0.932** |  | **0.6** | **0.365** |  |
| **P_0011** | **β-Estradiol 17α-Estradiol** | [**C00951 C02537**](http://www.genome.jp/dbget-bin/www_bget?cpd:C00951) | [**HMDB00151 HMDB00429**](http://www.hmdb.ca/metabolites/HMDB00151) | **0.9** | **0.698** |  | **1.5** | **0.014** | ***** | **0.9** | **0.461** |  | **0.8** | **0.178** |  | **1.2** | **0.239** |  | **0.9** | **0.741** |  | **0.8** | **0.236** |  |
| **C_0189** | **γ-Glu-Val-Gly** | **No ID** | **No ID** | **0.9** | **0.738** |  | **0.9** | **0.522** |  | **0.8** | **0.189** |  | **0.9** | **0.662** |  | **0.8** | **0.021** | ***** | **1.3** | **0.262** |  | **1.0** | **0.934** |  |

**Supplementary Table S4.** **Comparable analysis of metabolites in the skeletal muscles.**

For group comparisons, relative area ratios were calculated for each of the peaks in the skeletal muscles. The substances for which names were identified by the Kyoto Encyclopedia of Genes and Genomes (KEGG) and Human Metabolome Technologies Database (HMDB). The ID consists of the initial letter quantitative values. The ratios between the age-matched mouse group were calculated, as shown, for untreated *mdx* mice (DMD), DPSC-treated *mdx* mice (Treated-DMD), and C57BL/6 mice (wild type, WT); DMD vs. WT, and Treated DMD vs. WT or DMD. Data are presented as the mean, and statistical differences are shown as *p*-values (*^*^P* < 0.05, *^**^P* < 0.01, and ^***^*P <* 0.001), *t*-test, n = 3 for each group.

| **ID** | **HMT DB ^†^** | | | **Comparative Analysis** | | | | | | | | | | | | | | | | | | | | |
| --- | --- | --- | --- | --- | --- | --- | --- | --- | --- | --- | --- | --- | --- | --- | --- | --- | --- | --- | --- | --- | --- | --- | --- | --- |
|  | **Compound name** | **KEGG ID** | **HMDB ID** | **DMD P30 vs  WT P30** | | | **DMD P60 vs  WT P60** | | | **DMD P90 vs WT P90** | | | **Treated DMD P60 vs DMD P60** | | | **Treated DMD P60 vs WT P60** | | | **Treated DMD P90 vs DMD P90** | | | **Treated DMD P90 vs WT P90** | | |
|  |  |  |  | **Ratio ^¶^** | ***p*-value ^\|\|^** | | **Ratio ^¶^** | ***p*-value ^\|\|^** | | **Ratio ^¶^** | ***p*-value ^\|\|^** | | **Ratio ^¶^** | ***p*-value ^\|\|^** | | **Ratio ^¶^** | ***p*-value ^\|\|^** | | **Ratio ^¶^** | ***p*-value ^\|\|^** | | **Ratio ^¶^** | ***p*-value ^\|\|^** | |
| **C_0062** | **1-Methyl-4-imidazoleacetic acid** | [**C05828**](http://www.genome.jp/dbget-bin/www_bget?cpd:C05828) | [**HMDB02820**](http://www.hmdb.ca/metabolites/HMDB02820) | **0.5** | **0.217** |  | **1.6** | **0.165** |  | **1.9** | **0.084** |  | **0.4** | **0.046** | ***** | **0.6** | **0.182** |  | **0.8** | **0.576** |  | **1.6** | **0.328** |  |
| **C_0185** | **1-Methyladenosine** | [**C02494**](http://www.genome.jp/dbget-bin/www_bget?cpd:C02494) | [**HMDB03331**](http://www.hmdb.ca/metabolites/HMDB03331) | **1.1** | **0.713** |  | **1.2** | **0.126** |  | **1.1** | **0.342** |  | **0.8** | **0.035** | ***** | **1.0** | **0.680** |  | **1.1** | **N.A.** |  | **1.2** | **N.A.** |  |
| **C_0056** | **1-Methylnicotinamide** | [**C02918**](http://www.genome.jp/dbget-bin/www_bget?cpd:C02918) | [**HMDB00699**](http://www.hmdb.ca/metabolites/HMDB00699) | **1.6** | **0.008** | ****** | **2.5** | **0.054** |  | **2.7** | **0.009** | ****** | **1.6** | **0.077** |  | **4.0** | **0.025** | ***** | **1.3** | **0.132** |  | **3.5** | **0.006** | ****** |
| **A_0020** | **2-Hydroxyglutaric acid** | [**C02630,C01087,C03196**](http://www.genome.jp/dbget-bin/www_bget?cpd:C02630) | [**HMDB00606,HMDB00694**](http://www.hmdb.ca/metabolites/HMDB00606) | **0.7** | **0.049** | ***** | **0.7** | **0.206** |  | **1.1** | **0.635** |  | **1.0** | **0.819** |  | **0.7** | **0.260** |  | **0.8** | **0.322** |  | **0.9** | **0.202** |  |
| **A_0010** | **2-Hydroxyvaleric acid** |  | [**HMDB01863**](http://www.hmdb.ca/metabolites/HMDB01863) | **1.2** | **0.350** |  | **1.5** | **0.158** |  | **1.5** | **0.051** |  | **0.9** | **0.556** |  | **1.3** | **0.013** | ***** | **0.9** | **0.732** |  | **1.4** | **0.212** |  |
| **A_0036** | **3-Phosphoglyceric acid** | [**C00197**](http://www.genome.jp/dbget-bin/www_bget?cpd:C00197) | [**HMDB00807**](http://www.hmdb.ca/metabolites/HMDB00807) | **0.7** | **0.219** |  | **0.8** | **0.354** |  | **1.2** | **0.330** |  | **1.4** | **0.322** |  | **1.1** | **0.687** |  | **0.5** | **0.098** |  | **0.7** | **0.037** | ***** |
| **C_0065** | **4-Guanidinobutyric acid** | [**C01035**](http://www.genome.jp/dbget-bin/www_bget?cpd:C01035) | [**HMDB03464**](http://www.hmdb.ca/metabolites/HMDB03464) | **2.9** | **0.049** | ***** | **3.2** | **0.005** | ****** | **3.7** | **0.030** | ***** | **0.7** | **0.029** | ***** | **2.1** | **0.043** | ***** | **1.0** | **0.935** |  | **3.6** | **0.007** | ****** |
| **C_0086** | **5-Hydroxylysine** | [**C16741**](http://www.genome.jp/dbget-bin/www_bget?cpd:C16741) | [**HMDB00450**](http://www.hmdb.ca/metabolites/HMDB00450) | **0.8** | **0.196** |  | **0.6** | **0.026** | ***** | **0.8** | **0.184** |  | **1.2** | **0.282** |  | **0.8** | **0.056** |  | **1.9** | **0.047** | ***** | **1.5** | **0.112** |  |
| **A_0012** | **5-Oxoproline** | [**C01879**](http://www.genome.jp/dbget-bin/www_bget?cpd:C01879) | [**HMDB00267**](http://www.hmdb.ca/metabolites/HMDB00267) | **0.6** | **0.222** |  | **1.4** | **0.185** |  | **2.1** | **0.046** | ***** | **0.5** | **0.027** | ***** | **0.6** | **0.112** |  | **0.8** | **0.299** |  | **1.6** | **0.252** |  |
| **C_0175** | **Adenosine** | [**C00212**](http://www.genome.jp/dbget-bin/www_bget?cpd:C00212) | [**HMDB00050**](http://www.hmdb.ca/metabolites/HMDB00050) | **0.9** | **0.795** |  | **0.6** | **0.227** |  | **0.4** | **0.113** |  | **0.9** | **0.897** |  | **0.6** | **0.046** | ***** | **1.0** | **0.983** |  | **0.4** | **0.122** |  |
| **C_0123** | **ADMA** | [**C03626**](http://www.genome.jp/dbget-bin/www_bget?cpd:C03626) | [**HMDB01539**](http://www.hmdb.ca/metabolites/HMDB01539) | **0.9** | **0.709** |  | **1.1** | **0.427** |  | **1.9** | **0.031** | ***** | **0.9** | **0.232** |  | **1.0** | **0.694** |  | **0.9** | **0.736** |  | **1.8** | **0.057** |  |
| **A_0130** | **ADP-ribose** | [**C00301**](http://www.genome.jp/dbget-bin/www_bget?cpd:C00301) | [**HMDB01178**](http://www.hmdb.ca/metabolites/HMDB01178) | **1.0** | **0.854** |  | **0.8** | **0.049** | ***** | **1.3** | **0.003** | ****** | **1.4** | **0.217** |  | **1.2** | **0.418** |  | **0.9** | **0.480** |  | **1.2** | **0.316** |  |
| **N_0016** | **Arachidonic acid** | [**C00219**](http://www.genome.jp/dbget-bin/www_bget?cpd:C00219) | [**HMDB01043**](http://www.hmdb.ca/metabolites/HMDB01043) | **2.2** | **0.234** |  | **3.2** | **0.001** | ****** | **2.0** | **0.021** | ***** | **0.9** | **0.709** |  | **3.0** | **0.081** |  | **1.0** | **0.888** |  | **1.9** | **0.193** |  |
| **C_0095** | **Arg** | [**C00062,C00792**](http://www.genome.jp/dbget-bin/www_bget?cpd:C00062) | [**HMDB00517,HMDB03416**](http://www.hmdb.ca/metabolites/HMDB00517) | **1.0** | **0.894** |  | **1.3** | **0.442** |  | **1.8** | **0.147** |  | **0.7** | **0.317** |  | **0.9** | **0.613** |  | **1.0** | **0.948** |  | **1.9** | **0.011** | ***** |
| **C_0189** | **Argininosuccinic acid** | [**C03406**](http://www.genome.jp/dbget-bin/www_bget?cpd:C03406) | [**HMDB00052**](http://www.hmdb.ca/metabolites/HMDB00052) | **1.0** | **0.873** |  | **1.5** | **0.028** | ***** | **2.6** | **0.069** |  | **0.6** | **0.023** | ***** | **0.9** | **0.679** |  | **1.1** | **0.736** |  | **2.9** | **0.067** |  |
| **C_0049** | **Asn** | [**C00152,C01905,C16438**](http://www.genome.jp/dbget-bin/www_bget?cpd:C00152) | [**HMDB00168**](http://www.hmdb.ca/metabolites/HMDB00168) | **1.2** | **0.123** |  | **1.4** | **0.026** | ***** | **1.5** | **0.005** | ****** | **0.774** | **0.112** |  | **1.1** | **0.450** |  | **0.7** | **0.008** | ****** | **1.1** | **0.192** |  |
| **C_0052** | **Asp** | [**C00049,C00402,C16433**](http://www.genome.jp/dbget-bin/www_bget?cpd:C00049) | [**HMDB00191,HMDB06483**](http://www.hmdb.ca/metabolites/HMDB00191) | **0.8** | **0.485** |  | **1.3** | **0.089** |  | **1.6** | **0.064** |  | **1.1** | **0.765** |  | **1.4** | **0.213** |  | **0.9** | **0.400** |  | **1.4** | **0.018** | ***** |
| **A_0127** | **ATP** | [**C00002**](http://www.genome.jp/dbget-bin/www_bget?cpd:C00002) | [**HMDB00538**](http://www.hmdb.ca/metabolites/HMDB00538) | **0.5** | **0.045** | ***** | **0.6** | **0.008** | ****** | **0.9** | **0.313** |  | **1.3** | **0.370** |  | **0.7** | **0.205** |  | **0.7** | **0.049** | ***** | **0.6** | **0.002** | ****** |
| **C_0130** | **Carboxymethyllysine** |  |  | **0.8** | **0.279** |  | **0.6** | **0.001** | ****** | **1.1** | **0.673** |  | **1.6** | **0.036** | ***** | **1.0** | **0.929** |  | **1.4** | **0.002** | ****** | **1.6** | **0.137** |  |
| **C_0137** | **Carnosine** | [**C00386**](http://www.genome.jp/dbget-bin/www_bget?cpd:C00386) | [**HMDB00033**](http://www.hmdb.ca/metabolites/HMDB00033) | **0.9** | **0.160** |  | **0.8** | **0.294** |  | **0.7** | **0.004** | ***** | **1.0** | **0.841** |  | **0.8** | **0.259** |  | **1.2** | **0.042** |  | **0.9** | **0.125** |  |
| **C_0015** | **Choline** | [**C00114**](http://www.genome.jp/dbget-bin/www_bget?cpd:C00114) | [**HMDB00097**](http://www.hmdb.ca/metabolites/HMDB00097) | **0.9** | **0.509** |  | **1.2** | **0.390** |  | **2.9** | **0.001** | ******* | **0.9** | **0.367** |  | **1.0** | **0.901** |  | **0.4** | **0.008** | ****** | **1.3** | **0.139** |  |
| **N_0015** | ***cis*-5,8,11,14,17-Eicosapentaenoic acid Abietic acid** | [**C06428 C06087**](http://www.genome.jp/dbget-bin/www_bget?cpd:C06428) | [**HMDB01999**](http://www.hmdb.ca/metabolites/HMDB01999) | **1.6** | **0.428** |  | **1.2** | **0.411** |  | **1.2** | **0.503** |  | **0.9** | **0.617** |  | **1.1** | **0.845** |  | **1.8** | **0.016** | ***** | **2.1** | **0.036** | ***** |
| **A_0038** | **Citric acid** | [**C00158**](http://www.genome.jp/dbget-bin/www_bget?cpd:C00158) | [**HMDB00094**](http://www.hmdb.ca/metabolites/HMDB00094) | **0.8** | **0.254** |  | **0.8** | **0.031** | ***** | **1.0** | **0.871** |  | **1.2** | **0.034** | ***** | **0.9** | **0.197** |  | **1.0** | **0.895** |  | **1.0** | **0.971** |  |
| **A_0136** | **CMP-*N*-acetylneuraminate** | [**C00128**](http://www.genome.jp/dbget-bin/www_bget?cpd:C00128) | [**HMDB01176**](http://www.hmdb.ca/metabolites/HMDB01176) | **0.9** | **0.053** |  | **1.2** | **0.301** |  | **1.3** | **0.012** | ***** | **1.0** | **0.976** |  | **1.2** | **0.445** |  | **1.1** | **0.462** |  | **1.4** | **0.046** | ***** |
| **C_0050** | **Creatine** | [**C00300**](http://www.genome.jp/dbget-bin/www_bget?cpd:C00300) | [**HMDB00064**](http://www.hmdb.ca/metabolites/HMDB00064) | **0.9** | **0.218** |  | **0.9** | **0.660** |  | **1.2** | **0.100** |  | **1.0** | **0.878** |  | **0.9** | **0.496** |  | **1.0** | **0.639** |  | **1.2** | **0.024** | ***** |
| **A_0123** | **CTP** | [**C00063**](http://www.genome.jp/dbget-bin/www_bget?cpd:C00063) | [**HMDB00082**](http://www.hmdb.ca/metabolites/HMDB00082) | **0.9** | **0.512** |  | **1.1** | **0.392** |  | **1.7** | **0.037** | ***** | **1.1** | **0.557** |  | **1.2** | **0.412** |  | **0.6** | **0.043** | ***** | **1.1** | **0.207** |  |
| **C_0151** | **Cytidine** | [**C00475**](http://www.genome.jp/dbget-bin/www_bget?cpd:C00475) | [**HMDB00089**](http://www.hmdb.ca/metabolites/HMDB00089) | **1.2** | **0.171** |  | **1.8** | **0.010** | ****** | **2.1** | **0.004** | ****** | **0.7** | **0.182** |  | **1.4** | **0.368** |  | **0.9** | **0.167** |  | **1.8** | **0.008** | ****** |
| **C_0165** | **Dyphylline** | [**C07819**](http://www.genome.jp/dbget-bin/www_bget?cpd:C07819) |  | **1.9** | **0.014** | ***** | **1.4** | **0.073** |  | **1.2** | **0.459** |  | **1.3** | **0.104** |  | **1.8** | **0.003** | ****** | **0.8** | **0.125** |  | **0.9** | **0.716** |  |
| **C_0002** | **Ethanolamine** | [**C00189**](http://www.genome.jp/dbget-bin/www_bget?cpd:C00189) | [**HMDB00149**](http://www.hmdb.ca/metabolites/HMDB00149) | **1.1** | **0.492** |  | **1.3** | **0.209** |  | **1.7** | **0.020** | ***** | **0.8** | **0.461** |  | **1.1** | **0.760** |  | **0.8** | **0.038** | ***** | **1.3** | **0.179** |  |
| **A_0018** | **Ethanolamine phosphate** | [**C00346**](http://www.genome.jp/dbget-bin/www_bget?cpd:C00346) | [**HMDB00224**](http://www.hmdb.ca/metabolites/HMDB00224) | **1.9** | **0.024** | ***** | **2.5** | **0.002** | ******** | **2.9** | **0.001** | ****** | **1.0** | **0.981** |  | **2.5** | **1.0E-04** | ******* | **1.0** | **1.000** |  | **2.9** | **0.001** | ****** |
| **A_0007** | **Fumaric acid** | [**C00122**](http://www.genome.jp/dbget-bin/www_bget?cpd:C00122) | [**HMDB00134**](http://www.hmdb.ca/metabolites/HMDB00134) | **0.8** | **0.162** |  | **0.6** | **0.012** | ***** | **1.1** | **0.705** |  | **1.1** | **0.492** |  | **0.7** | **0.019** | ***** | **1.0** | **0.913** |  | **1.1** | **0.378** |  |
| **C_0068** | **Gln** | [**C00064,C00303,C00819**](http://www.genome.jp/dbget-bin/www_bget?cpd:C00064) | [**HMDB00641,HMDB03423**](http://www.hmdb.ca/metabolites/HMDB00641) | **1.1** | **0.101** |  | **1.2** | **0.196** |  | **1.6** | **0.016** | ***** | **0.9** | **0.611** |  | **1.1** | **0.353** |  | **0.8** | **0.044** | ***** | **1.2** | **0.152** |  |
| **C_0048** | **Gly-Gly** | [**C02037**](http://www.genome.jp/dbget-bin/www_bget?cpd:C02037) | [**HMDB11733**](http://www.hmdb.ca/metabolites/HMDB11733) | **1.2** | **0.063** |  | **1.7** | **0.044** | ***** | **2.0** | **0.042** | ***** | **0.8** | **0.243** |  | **1.3** | **0.387** |  | **0.9** | **0.401** |  | **1.7** | **0.070** |  |
| **C_0010** | **Glycerol** | [**C00116**](http://www.genome.jp/dbget-bin/www_bget?cpd:C00116) | [**HMDB00131**](http://www.hmdb.ca/metabolites/HMDB00131) | **1.4** | **0.013** | ***** | **1.0** | **0.918** |  | **1.0** | **0.904** |  | **1.2** | **0.472** |  | **1.2** | **0.134** |  | **0.6** | **0.033** | ***** | **0.7** | **0.364** |  |
| **C_0168** | **Glycerophosphocholine** | [**C00670**](http://www.genome.jp/dbget-bin/www_bget?cpd:C00670) | [**HMDB00086**](http://www.hmdb.ca/metabolites/HMDB00086) | **1.6** | **0.035** | ***** | **1.9** | **0.048** | ***** | **2.8** | **0.005** | ****** | **0.8** | **0.280** |  | **1.5** | **0.078** |  | **1.0** | **0.894** |  | **2.7** | **0.024** | ***** |
| **A_0128** | **GTP** | [**C00044**](http://www.genome.jp/dbget-bin/www_bget?cpd:C00044) | [**HMDB01273**](http://www.hmdb.ca/metabolites/HMDB01273) | **0.9** | **0.387** |  | **1.0** | **0.936** |  | **1.6** | **0.030** | ***** | **1.0** | **0.938** |  | **1.0** | **0.922** |  | **0.8** | **0.093** |  | **1.2** | **0.058** |  |
| **C_0028** | **Guanidoacetic acid** | [**C00581**](http://www.genome.jp/dbget-bin/www_bget?cpd:C00581) | [**HMDB00128**](http://www.hmdb.ca/metabolites/HMDB00128) | **1.5** | **0.079** |  | **2.1** | **0.120** |  | **2.8** | **0.003** | ****** | **0.8** | **0.498** |  | **1.7** | **0.022** | ***** | **0.9** | **0.228** |  | **2.5** | **0.007** | ****** |
| **C_0076** | **His** | [**C00135,C00768,C06419**](http://www.genome.jp/dbget-bin/www_bget?cpd:C00135) | [**HMDB00177**](http://www.hmdb.ca/metabolites/HMDB00177) | **0.8** | **0.068** |  | **0.9** | **0.485** |  | **1.1** | **0.375** |  | **0.9** | **0.356** |  | **0.8** | **0.213** |  | **0.8** | **0.004** | ****** | **0.8** | **0.186** |  |
| **C_0023** | **Histamine** | [**C00388**](http://www.genome.jp/dbget-bin/www_bget?cpd:C00388) | [**HMDB00870**](http://www.hmdb.ca/metabolites/HMDB00870) | **0.7** | **0.075** |  | **0.8** | **0.015** | ***** | **1.0** | **0.972** |  | **1.3** | **0.024** | ***** | **1.0** | **0.454** |  | **1.4** | **0.018** | ***** | **1.4** | **0.059** |  |
| **C_0146** | **Homocarnosine** | [**C00884**](http://www.genome.jp/dbget-bin/www_bget?cpd:C00884) | [**HMDB00745**](http://www.hmdb.ca/metabolites/HMDB00745) | **0.6** | **0.009** | ****** | **0.4** | **0.037** | ***** | **0.4** | **0.004** | ****** | **1.0** | **0.678** |  | **0.4** | **0.039** | ***** | **1.2** | **0.043** | ***** | **0.5** | **0.011** | ***** |
| **C_0045** | **Hydroxyproline** | [**C01157**](http://www.genome.jp/dbget-bin/www_bget?cpd:C01157) | [**HMDB00725**](http://www.hmdb.ca/metabolites/HMDB00725) | **1.3** | **0.101** |  | **0.9** | **0.383** |  | **1.0** | **0.728** |  | **0.9** | **0.594** |  | **0.8** | **0.180** |  | **0.8** | **0.120** |  | **0.8** | **0.002** | ****** |
| **C_0055** | **Hypoxanthine** | [**C00262**](http://www.genome.jp/dbget-bin/www_bget?cpd:C00262) | [**HMDB00157**](http://www.hmdb.ca/metabolites/HMDB00157) | **2.2** | **0.082** |  | **2.2** | **0.225** |  | **1.3** | **0.527** |  | **0.8** | **0.633** |  | **1.7** | **0.272** |  | **1.8** | **0.066** |  | **2.2** | **0.018** | ***** |
| **C_0046** | **Ile** | [**C00407,C06418,C16434**](http://www.genome.jp/dbget-bin/www_bget?cpd:C00407) | [**HMDB00172**](http://www.hmdb.ca/metabolites/HMDB00172) | **0.9** | **0.035** | ***** | **1.1** | **0.547** |  | **0.9** | **0.636** |  | **0.9** | **0.526** |  | **1.0** | **0.886** |  | **1.2** | **0.272** |  | **1.1** | **0.334** |  |
| **A_0092** | **IMP** | [**C00130**](http://www.genome.jp/dbget-bin/www_bget?cpd:C00130) | [**HMDB00175**](http://www.hmdb.ca/metabolites/HMDB00175) | **2.5** | **0.069** |  | **1.8** | **0.144** |  | **1.2** | **0.737** |  | **0.6** | **0.183** |  | **1.0** | **0.988** |  | **2.5** | **0.032** | ***** | **3.0** | **0.005** | ****** |
| **C_0176** | **Inosine** | [**C00294**](http://www.genome.jp/dbget-bin/www_bget?cpd:C00294) | [**HMDB00195**](http://www.hmdb.ca/metabolites/HMDB00195) | **1.5** | **0.071** |  | **1.4** | **0.388** |  | **0.9** | **0.719** |  | **0.9** | **0.669** |  | **1.2** | **0.506** |  | **1.5** | **0.013** | ***** | **1.4** | **0.129** |  |
| **A_0011** | **Isethionic acid** | [**C05123**](http://www.genome.jp/dbget-bin/www_bget?cpd:C05123) | [**HMDB03903**](http://www.hmdb.ca/metabolites/HMDB03903) | **0.9** | **0.480** |  | **1.0** | **0.863** |  | **1.4** | **0.048** | ***** | **1.1** | **0.542** |  | **1.0** | **0.757** |  | **1.0** | **0.961** |  | **1.4** | **0.056** |  |
| **C_0069** | **Lys** | [**C00047,C00739,C16440**](http://www.genome.jp/dbget-bin/www_bget?cpd:C00047) | [**HMDB00182,HMDB03405**](http://www.hmdb.ca/metabolites/HMDB00182) | **0.9** | **0.837** |  | **1.2** | **0.627** |  | **2.0** | **0.121** |  | **0.7** | **0.400** |  | **0.9** | **0.557** |  | **1.0** | **0.928** |  | **2.0** | **0.011** | ***** |
| **A_0014** | **Malic acid** | [**C00149,C00497,C00711**](http://www.genome.jp/dbget-bin/www_bget?cpd:C00149) | [**HMDB00156,HMDB00744**](http://www.hmdb.ca/metabolites/HMDB00156) | **0.9** | **0.676** |  | **0.8** | **0.002** | ****** | **1.0** | **0.870** |  | **1.0** | **0.668** |  | **0.7** | **0.027** | ***** | **1.2** | **0.149** |  | **1.2** | **0.094** |  |
| **A_0065** | ***myo*-Inositol 1-phosphate *myo*-Inositol 3-phosphate** | [**C01177 C04006**](http://www.genome.jp/dbget-bin/www_bget?cpd:C01177) | [**HMDB00213 HMDB06814**](http://www.hmdb.ca/metabolites/HMDB00213) | **0.8** | **0.247** |  | **1.1** | **0.155** |  | **1.7** | **0.036** | ***** | **1.4** | **0.221** |  | **1.5** | **0.160** |  | **0.9** | **0.365** |  | **1.5** | **0.031** | ***** |
| **C_0014** | ***N*,*N*-Dimethylglycine** | [**C01026**](http://www.genome.jp/dbget-bin/www_bget?cpd:C01026) | [**HMDB00092**](http://www.hmdb.ca/metabolites/HMDB00092) | **1.2** | **0.042** | ***** | **1.2** | **0.330** |  | **1.2** | **0.180** |  | **0.9** | **0.409** |  | **1.0** | **0.933** |  | **1.0** | **0.744** |  | **1.2** | **0.153** |  |
| **C_0094** | ***N*^5^-Ethylglutamine** | [**C01047**](http://www.genome.jp/dbget-bin/www_bget?cpd:C01047) |  | **0.9** | **0.559** |  | **1.3** | **0.030** | ***** | **1.0** | **0.898** |  | **0.8** | **0.203** |  | **1.1** | **0.522** |  | **1.0** | **0.782** |  | **0.9** | **0.804** |  |
| **A_0138** | **NAD^+^** | [**C00003**](http://www.genome.jp/dbget-bin/www_bget?cpd:C00003) | [**HMDB00902**](http://www.hmdb.ca/metabolites/HMDB00902) | **0.6** | **0.016** | ***** | **0.6** | **0.004** | ****** | **0.9** | **0.311** |  | **0.9** | **0.625** |  | **0.6** | **0.025** | ***** | **0.7** | **0.142** |  | **0.6** | **0.013** | ***** |
| **C_0037** | **Nicotinamide** | [**C00153**](http://www.genome.jp/dbget-bin/www_bget?cpd:C00153) | [**HMDB01406**](http://www.hmdb.ca/metabolites/HMDB01406) | **1.2** | **0.200** |  | **1.1** | **0.333** |  | **1.3** | **0.273** |  | **1.0** | **0.883** |  | **1.1** | **0.534** |  | **1.4** | **0.102** |  | **1.9** | **0.010** | ****** |
| **C_0082** | ***O*-Acetylhomoserine 2-Aminoadipic acid** | [**C01077 C00956**](http://www.genome.jp/dbget-bin/www_bget?cpd:C01077) | [**HMDB00510**](http://www.hmdb.ca/metabolites/) | **1.2** | **0.030** | ***** | **1.6** | **0.006** | ****** | **2.3** | **4.0E-04** | ******* | **1.0** | **0.998** |  | **1.5** | **0.007** | ****** | **0.8** | **0.915** |  | **2.0** | **9.0E-04** | ******* |
| **P_0017** | **Oleoyl ethanolamine** |  |  | **0.5** | **0.171** |  | **1.1** | **0.679** |  | **2.1** | **0.023** | ***** | **1.9** | **0.313** |  | **2.0** | **0.281** |  | **0.5** | **0.016** | ***** | **1.0** | **0.945** |  |
| **C_0051** | **Ornithine** | [**C00077,C00515,C01602**](http://www.genome.jp/dbget-bin/www_bget?cpd:C00077) | [**HMDB00214,HMDB03374**](http://www.hmdb.ca/metabolites/HMDB00214) | **1.0** | **0.931** |  | **1.0** | **0.828** |  | **0.8** | **0.456** |  | **1.0** | **0.936** |  | **1.1** | **0.817** |  | **2.4** | **0.038** | ***** | **2.0** | **0.044** | ***** |
| **A_0017** | ***p*-Toluic acid *m*-Toluic acid *o*-Toluic acid** | [**C01454 C07211 C07215**](http://www.genome.jp/dbget-bin/www_bget?cpd:C01454) |  | **0.7** | **0.218** |  | **0.8** | **0.627** |  | **1.0** | **0.944** |  | **1.1** | **0.661** |  | **1.0** | **0.871** |  | **0.5** | **0.032** | ***** | **0.5** | **0.093** |  |
| **P_0010** | **Palmitoylethanolamide** |  | [**HMDB02100**](http://www.hmdb.ca/metabolites/HMDB02100) | **0.8** | **0.459** |  | **1.2** | **0.564** |  | **4.8** | **0.109** |  | **8.0** | **0.198** |  | **9.4** | **0.192** |  | **0.2** | **0.115** |  | **1.2** | **0.449** |  |
| **A_0050** | **Pantothenic acid** | [**C00864**](http://www.genome.jp/dbget-bin/www_bget?cpd:C00864) | [**HMDB00210**](http://www.hmdb.ca/metabolites/HMDB00210) | **4.8** | **0.001** | ****** | **3.1** | **0.004** | ****** | **4.4** | **0.011** | ***** | **0.729** | **0.291** |  | **2.3** | **0.0944** |  | **1.6** | **0.0687** |  | **6.8** | **1.0E-05** | ******** |
| **A_0048** | **Phosphocreatine** | [**C02305**](http://www.genome.jp/dbget-bin/www_bget?cpd:C02305) | [**HMDB01511**](http://www.hmdb.ca/metabolites/HMDB01511) | **0.3** | **0.105** |  | **0.2** | **0.232** |  | **0.7** | **0.292** |  | **2.8** | **0.248** |  | **0.7** | **0.612** |  | **0.3** | **0.164** |  | **0.2** | **0.011** | ***** |
| **C_0102** | **Phosphorylcholine** | [**C00588**](http://www.genome.jp/dbget-bin/www_bget?cpd:C00588) | [**HMDB01565**](http://www.hmdb.ca/metabolites/HMDB01565) | **1.5** | **0.018** | ***** | **1.4** | **0.055** |  | **1.2** | **0.163** |  | **1.0** | **0.878** |  | **1.3** | **0.375** |  | **0.9** | **0.300** |  | **1.1** | **0.556** |  |
| **C_0043** | **Pipecolic acid** | [**C00408**](http://www.genome.jp/dbget-bin/www_bget?cpd:C00408) | [**HMDB00070,HMDB00716,HMDB05960**](http://www.hmdb.ca/metabolites/HMDB00070) | **1.0** | **0.632** |  | **1.1** | **0.470** |  | **1.2** | **0.338** |  | **1.2** | **0.520** |  | **1.3** | **0.354** |  | **1.0** | **0.807** |  | **1.3** | **0.049** | ***** |
| **C_0026** | **Pro** | [**C00148,C00763,C16435**](http://www.genome.jp/dbget-bin/www_bget?cpd:C00148) | [**HMDB00162,HMDB03411**](http://www.hmdb.ca/metabolites/HMDB00162) | **0.9** | **0.451** |  | **1.0** | **0.843** |  | **1.0** | **0.598** |  | **0.8** | **0.310** |  | **0.8** | **0.425** |  | **0.9** | **0.048** | ***** | **0.9** | **0.243** |  |
| **C_0005** | **Putrescine** | [**C00134**](http://www.genome.jp/dbget-bin/www_bget?cpd:C00134) | [**HMDB01414**](http://www.hmdb.ca/metabolites/HMDB01414) | **2.2** | **0.035** | ***** | **3.2** | **0.008** | ****** | **3.4** | **0.011** | ***** | **0.9** | **0.795** |  | **3.0** | **0.002** | ****** | **1.1** | **0.586** |  | **3.8** | **0.006** | ****** |
| **C_0158** | **Pyridoxamine 5'-phosphate** | [**C00647**](http://www.genome.jp/dbget-bin/www_bget?cpd:C00647) | [**HMDB01555**](http://www.hmdb.ca/metabolites/HMDB01555) | **0.7** | **0.027** | ***** | **0.6** | **0.130** |  | **0.9** | **0.671** |  | **1.5** | **0.250** |  | **0.9** | **0.349** |  | **0.8** | **0.298** |  | **0.7** | **0.178** |  |
| **A_0053** | **Ribulose 5-phosphate** | [**C00199,C01101**](http://www.genome.jp/dbget-bin/www_bget?cpd:C00199) | [**HMDB00618**](http://www.hmdb.ca/metabolites/HMDB00618) | **1.8** | **0.151** |  | **1.8** | **0.016** | ***** | **1.9** | **0.067** |  | **0.9** | **0.642** |  | **1.7** | **0.114** |  | **1.2** | **0.270** |  | **2.3** | **0.020** | ***** |
| **C_0210** | ***S*-Adenosylmethionine** | [**C00019**](http://www.genome.jp/dbget-bin/www_bget?cpd:C00019) | [**HMDB01185**](http://www.hmdb.ca/metabolites/HMDB01185) | **1.1** | **0.399** |  | **1.2** | **0.211** |  | **1.4** | **0.087** |  | **0.8** | **0.212** |  | **1.0** | **0.973** |  | **1.1** | **0.308** |  | **1.6** | **0.031** | ***** |
| **C_0006** | **Sarcosine** | [**C00213**](http://www.genome.jp/dbget-bin/www_bget?cpd:C00213) | [**HMDB00271**](http://www.hmdb.ca/metabolites/HMDB00271) | **1.0** | **0.982** |  | **0.8** | **0.321** |  | **0.8** | **0.142** |  | **0.9** | **0.614** |  | **0.7** | **0.027** | ***** | **1.4** | **0.224** |  | **1.1** | **0.673** |  |
| **C_0124** | **SDMA** |  | [**HMDB03334**](http://www.hmdb.ca/metabolites/HMDB03334) | **1.0** | **0.856** |  | **1.2** | **0.260** |  | **2.4** | **0.033** | ***** | **0.7** | **0.183** |  | **0.9** | **0.537** |  | **0.9** | **0.516** |  | **2.1** | **0.049** | ***** |
| **P_0011** | **Sphinganine** | [**C00836**](http://www.genome.jp/dbget-bin/www_bget?cpd:C00836) | [**HMDB00269**](http://www.hmdb.ca/metabolites/HMDB00269) | **1.4** | **0.046** | ***** | **1.8** | **2.0E-04** | ******* | **2.0** | **0.007** | ****** | **0.8** | **0.133** |  | **1.5** | **0.016** | ***** | **1.0** | **0.817** |  | **1.8** | **0.025** | ***** |
| **P_0009** | **Sphingosine** | [**C00319**](http://www.genome.jp/dbget-bin/www_bget?cpd:C00319) | [**HMDB00252**](http://www.hmdb.ca/metabolites/HMDB00252) | **1.6** | **0.027** | ***** | **1.9** | **0.002** | ****** | **1.8** | **0.029** | ***** | **0.8** | **0.196** |  | **1.5** | **0.068** |  | **0.9** | **0.852** |  | **1.6** | **0.087** |  |
| **C_0063** | **Stachydrine** | [**C10172**](http://www.genome.jp/dbget-bin/www_bget?cpd:C10172) | [**HMDB04827**](http://www.hmdb.ca/metabolites/HMDB04827) | **1.1** | **0.610** |  | **1.4** | **0.027** | ***** | **1.1** | **0.815** |  | **0.9** | **0.263** |  | **1.2** | **0.116** |  | **1.1** | **0.266** |  | **1.2** | **0.452** |  |
| **A_0009** | **Succinic acid** | [**C00042**](http://www.genome.jp/dbget-bin/www_bget?cpd:C00042) | [**HMDB00254**](http://www.hmdb.ca/metabolites/HMDB00254) | **0.8** | **0.554** |  | **0.8** | **0.158** |  | **1.1** | **0.667** |  | **0.7** | **0.097** |  | **0.6** | **0.005** | ****** | **0.8** | **0.349** |  | **0.9** | **0.288** |  |
| **C_0039** | **Taurine** | [**C00245**](http://www.genome.jp/dbget-bin/www_bget?cpd:C00245) | [**HMDB00251**](http://www.hmdb.ca/metabolites/HMDB00251) | **0.8** | **0.070** |  | **0.9** | **0.029** | ***** | **1.0** | **0.494** |  | **1.0** | **0.885** |  | **0.9** | **0.216** |  | **1.1** | **0.041** | ***** | **1.1** | **0.020** | ***** |
| **N_0037** | **Taurocholic acid** | [**C05122**](http://www.genome.jp/dbget-bin/www_bget?cpd:C05122) | [**HMDB00036**](http://www.hmdb.ca/metabolites/HMDB00036) | **2.4** | **0.604** |  | **26** | **N.A.** |  | **6.7** | **0.217** |  | **0.3** | **0.586** |  | **6.6** | **N.A.** |  | **1.0** | **0.949** |  | **6.9** | **0.043** | ***** |
| **C_0090** | **Taurocyamine** | [**C01959**](http://www.genome.jp/dbget-bin/www_bget?cpd:C01959) | [**HMDB03584**](http://www.hmdb.ca/metabolites/HMDB03584) | **1.7** | **0.006** | ****** | **1.6** | **0.224** |  | **1.9** | **0.003** | ****** | **1.0** | **0.889** |  | **1.6** | **0.008** | ****** | **0.8** | **0.071** |  | **1.6** | **0.015** | ***** |
| **A_0015** | **Threonic acid** | [**C01620**](http://www.genome.jp/dbget-bin/www_bget?cpd:C01620) | [**HMDB00943**](http://www.hmdb.ca/metabolites/HMDB00943) | **2.0** | **0.010** | ****** | **2.2** | **1.0E-05** | ******** | **3.3** | **0.003** | ******* | **0.9** | **0.277** |  | **1.9** | **5.0E-04** | ******** | **1.2** | **0.473** |  | **3.8** | **1.0E-05** | ******** |
| **C_0101** | **Tyr** | [**C00082,C01536,C06420**](http://www.genome.jp/dbget-bin/www_bget?cpd:C00082) | [**HMDB00158**](http://www.hmdb.ca/metabolites/HMDB00158) | **0.9** | **0.508** |  | **1.1** | **0.733** |  | **0.8** | **0.016** | ***** | **1.1** | **0.730** |  | **1.2** | **0.552** |  | **1.1** | **0.399** |  | **0.9** | **0.064** |  |
| **A_0131** | **UDP-glucose UDP-galactose** | [**C00029 C00052**](http://www.genome.jp/dbget-bin/www_bget?cpd:C00029) | [**HMDB00286 HMDB00302**](http://www.hmdb.ca/metabolites/HMDB00286) | **1.3** | **0.160** |  | **1.6** | **0.057** |  | **1.8** | **0.036** | ***** | **0.8** | **0.316** |  | **1.3** | **0.278** |  | **0.8** | **0.178** |  | **1.4** | **0.192** |  |
| **A_0135** | **UDP-*N*-acetylgalactosamine UDP-*N*-acetylglucosamine** | [**C00203 C00043**](http://www.genome.jp/dbget-bin/www_bget?cpd:C00203) | [**HMDB00304 HMDB00290**](http://www.hmdb.ca/metabolites/HMDB00304) | **1.7** | **0.004** | ****** | **2.4** | **0.085** |  | **2.9** | **8.0E-04** | ******* | **0.8** | **0.392** |  | **1.9** | **0.075** |  | **0.9** | **0.177** |  | **2.6** | **0.003** | ****** |
| **A_0027** | **Uric acid** | [**C00366**](http://www.genome.jp/dbget-bin/www_bget?cpd:C00366) | [**HMDB00289**](http://www.hmdb.ca/metabolites/HMDB00289) | **1.7** | **0.005** |  | **2.2** | **0.000** | ******* | **2.4** | **0.004** | ****** | **1.1** | **0.614** |  | **2.3** | **0.000** | ******** | **0.8** | **0.006** | ****** | **2.1** | **0.032** | ***** |
| **C_0152** | **Uridine** | [**C00299**](http://www.genome.jp/dbget-bin/www_bget?cpd:C00299) | [**HMDB00296**](http://www.hmdb.ca/metabolites/HMDB00296) | **1.2** | **0.157** |  | **1.9** | **0.002** | ****** | **1.4** | **N.A.** |  | **0.8** | **0.129** |  | **1.4** | **0.155** |  | **1.0** | **0.851** |  | **1.4** | **N.A.** |  |
| **C_0061** | **Urocanic acid** | [**C00785**](http://www.genome.jp/dbget-bin/www_bget?cpd:C00785) | [**HMDB00301**](http://www.hmdb.ca/metabolites/HMDB00301) | **0.5** | **0.005** | ****** | **0.5** | **0.006** | ****** | **0.4** | **4.0E-04** | ******* | **1.0** | **0.998** |  | **0.5** | **0.007** | ****** | **1.1** | **0.915** |  | **0.5** | **0.001** | ******* |
| **A_0124** | **UTP** | [**C00075**](http://www.genome.jp/dbget-bin/www_bget?cpd:C00075) | [**HMDB00285**](http://www.hmdb.ca/metabolites/HMDB00285) | **0.8** | **0.534** |  | **1.3** | **0.070** |  | **1.7** | **0.015** | ***** | **1.0** | **0.904** |  | **1.3** | **0.367** |  | **0.7** | **0.027** | ***** | **1.2** | **0.154** |  |
| **C_0035** | **XC0013 Anserine_divalent** | [**C01262**](http://www.genome.jp/dbget-bin/www_bget?cpd:) | [**HMDB00194**](http://www.hmdb.ca/metabolites/) | **0.6** | **0.014** | ***** | **0.5** | **0.014** | ***** | **0.5** | **7.4E-04** | ******* | **1.0** | **0.622** |  | **0.5** | **0.021** | ***** | **1.2** | **0.031** | ***** | **0.6** | **0.003** | ****** |
| **C_0042** | **XC0016** |  |  | **2.6** | **0.046** | ***** | **1.9** | **0.049** | ***** | **2.3** | **0.008** | ****** | **0.9** | **0.440** |  | **1.6** | **0.035** | ***** | **1.0** | **0.959** |  | **2.3** | **0.003** | ****** |
| **C_0199** | **XC0132** |  |  | **1.8** | **0.048** | ***** | **2.6** | **0.067** |  | **2.5** | **0.057** |  | **0.8** | **0.408** |  | **2.1** | **0.035** | ***** | **1.0** | **0.865** |  | **2.4** | **0.002** | ****** |
| **C_0009** | **β-Ala** | [**C00099**](http://www.genome.jp/dbget-bin/www_bget?cpd:C00099) | [**HMDB00056**](http://www.hmdb.ca/metabolites/HMDB00056) | **1.2** | **0.212** |  | **0.8** | **0.221** |  | **1.1** | **0.333** |  | **1.0** | **0.844** |  | **0.7** | **0.051** |  | **1.2** | **0.110** |  | **1.3** | **0.047** | ***** |
| **P_0006** | **β-Estradiol 17α-Estradiol** | [**C00951 C02537**](http://www.genome.jp/dbget-bin/www_bget?cpd:C00951) | [**HMDB00151 HMDB00429**](http://www.hmdb.ca/metabolites/HMDB00151) | **0.6** | **0.013** | ***** | **0.7** | **0.246** |  | **1.1** | **0.680** |  | **1.1** | **0.224** |  | **0.8** | **0.375** |  | **1.2** | **0.293** |  | **1.3** | **0.131** |  |
| **C_0066** | **γ-Butyrobetaine** | [**C01181**](http://www.genome.jp/dbget-bin/www_bget?cpd:C01181) | [**HMDB01161**](http://www.hmdb.ca/metabolites/HMDB01161) | **1.0** | **0.905** |  | **1.2** | **0.233** |  | **1.3** | **0.071** |  | **0.8** | **0.224** |  | **1.0** | **0.773** |  | **1.1** | **0.505** |  | **1.4** | **0.019** | ***** |

**Supplementary Table S5. Upstream regulator analysis using IPA**

The plasma samples derived from 60-day-old wild type (WT), untreated *mdx* mice, and DPSC-treated *mdx* mice (treated-*mdx*) were compared using “Upstream regulator” analysis by IPA and were listed by activated upstream regulators, for both down-regulated factors (predicted activation state; Inhibited, activation z-score; < -2.0), and up-regulated factors (predicted activation state; Activated, activation z-score; > 2.0). The target molecules in the dataset column indicate the number of target factors registered in the database for that regulator.

|  | Upstream Regulator | Predicted Activation State | *p*-value of overlap | Activation z-score | # Target molecules in dataset | Target molecules in dataset |
| --- | --- | --- | --- | --- | --- | --- |
| WT vs. *mdx* | LEP | Inhibited | 3.48E-01 | -2.071 | 8 | 5-hydroxytryptamine, ATP, cholesterol, creatinine, D-sphingosine, glutathione, glycerol, progesterone |
|  | NOS3 | Inhibited | 5.90E-02 | -2.412 | 6 | ATP, cholesterol, citrulline, creatinine, L-arginine, progesterone |
|  | 3-nitropropionic acid | Inhibited | 1.46E-03 | -2.187 | 8 | AMP, ATP, GABA, GDP, glutathione, GTP, L-glutamic acid, phosphocreatine |
|  | LDL | Activated | 1.03E-01 | 2.423 | 6 | cholesterol, D-sphingosine, glutathione, oleic acid, phosphorylcholine, progesterone |
|  | BHMT | Activated | 1.11E-02 | 2.204 | 5 | cholesterol, choline, phosphorylcholine, S-adenosylmethionine, sn-glycero-3-phosphocholine |
| *mdx* vs. MSC-treated *mdx* | methamphetamine | Inhibited | 2.79E-02 | -2.176 | 5 | 4-hydroxy-3-methoxyphenylacetic acid, 5-hydroxytryptamine, creatinine, GABA, glutathione |
|  | BHMT | Activated | 1.28E-02 | 2.204 | 5 | cholesterol, choline, phosphorylcholine, S-adenosylmethionine, sn-glycero-3-phosphocholine |
|  | UCP1 | Activated | 1.28E-04 | 2.496 | 13 | arachidonic acid, ATP, cholesterol, dl-cystathionine, glycine, L-glutamic acid, L-serine, L-threonine, lauric acid, myristic acid, oleic acid, spermidine, stearic acid |
|  | NOS1 | Activated | 1.03E-01 | 2.236 | 5 | citrulline, creatinine, GABA, L-arginine, L-glutamic acid |
|  | GNRH1 | Activated | 8.35E-02 | 2.188 | 5 | GABA, L-glutamic acid, progesterone, testosterone, urea |
